# HDGS-Net: nucleosome occupancy prediction based on a hybrid dilated gated separable convolutional neural network

**DOI:** 10.1186/s12864-026-12523-2

**Published:** 2026-01-24

**Authors:** Fuquan Shi, Meizhi Wang, Zhixia Teng, Lu Cai, Guoqing Liu, Yongqiang Xing, Xiangjun Cui, Guojun Liu, Zhihua Yang, Hu Meng

**Affiliations:** 1 Inner Mongolia Key Laboratory of Life Health and Bioinformatics, College of Life Science and Technology, Inner Mongolia University of Science and Technology, Baotou, 014010 China; 2 College of Information and Computer Engineering, Northeast Forestry University, Harbin, China; 3 Present Address: Baotou, China

**Keywords:** Nucleosome positioning, Deep learning, Hybrid convolution, Sequence dependence

## Abstract

Nucleosome positioning plays a central role in chromatin organization and gene regulation, yet its accurate computational prediction remains challenging. This study introduces a Hybrid Dilated Gated Separable Convolutional Neural Network (HDGS-Net), which integrates dilated convolution, gated convolution, and depthwise separable convolution to achieve continuous prediction of in vitro nucleosome occupancy at single-base resolution across the entire Saccharomyces cerevisiae genome. On benchmark datasets, HDGS-Net attained an average Pearson correlation coefficient of 0.87, outperforming conventional methods and demonstrating excellent cross-chromosome generalization capability. Sequence analysis confirms that DNA dinucleotide physical properties dominate nucleosome positioning, with AT-rich sequences inhibiting binding and GC-rich sequences promoting binding. Analysis of transcription start regions verifies that flanking nucleosome sequence features are highly conserved across different chromatin environments, supporting the universal regulatory role of sequence preference. Cross-species analysis demonstrates that the guiding efficacy of DNA sequence on nucleosome positioning varies among species, showing quantitatively decreasing contributions in Caenorhabditis elegans, Saccharomyces cerevisiae, and Schizosaccharomyces pombe. This study provides a high-accuracy predictive tool for investigating dynamic nucleosome positioning.

**Supplementary Information:**

The online version contains supplementary material available at 10.1186/s12864-026-12523-2.

## Introduction

Nucleosomes, the fundamental structural units of eukaryotic chromatin, are formed by approximately 147 bp of DNA wrapped around a histone octamer in 1.7 left-handed superhelical turns. This structure is stabilized by linker histone H1 and connected by 10–100 bp linker DNA to form higher-order chromatin structures [1–8]. This precise assembly mechanism not only underlies genome compaction but also plays a central regulatory role in key cellular processes such as DNA replication [9–16], gene transcription [17–22], RNA splicing [23–25], DNA repair [26, 27], and genetic recombination [28–31] by dynamically regulating DNA accessibility [32–35]. Precisely mapping nucleosome positions at a genome-wide level has become a crucial prerequisite for deciphering epigenetic regulatory mechanisms.

Nucleosome positioning is co-regulated by multiple factors, including the DNA sequence itself, chromatin remodelers, transcription factors, DNA methylation, and histone modifications [4, 19, 36–51]. Despite the complexity of the regulatory network, numerous studies have confirmed that the intrinsic structural preferences of DNA sequences are the dominant factor determining nucleosome organization [6, 52–55]. Segal et al. proposed that DNA sequences explain approximately 50% of in vivo nucleosome positions [6]. Kaplan et al. further clarified that sequence preference is a key determinant of nucleosome assembly [52]. Ioshikhes et al. reported that about 75% of nucleosome positioning can be defined by sequence features [53]. These studies collectively establish the foundational role of sequence dependence in nucleosome positioning.

With the development of high-throughput sequencing technologies, such as MNase-seq, DNase-seq, ChIP-seq, ATAC-seq, NOME-seq, and chemical cleavage [19, 34, 52, 56–67], high-resolution nucleosome maps have been generated in multiple model organisms, including Saccharomyces cerevisiae [52, 59, 68, 69], Caenorhabditis elegans [70, 71], Drosophila [72, 73], and humans [19, 74, 75]. These data have been systematically integrated into the NucMap database [76], providing an essential resource for predictive methods. However, existing experimental techniques still exhibit significant limitations: methods like DNase-seq and ATAC-seq struggle to achieve single-base resolution; chemical cleavage techniques, while offering higher resolution, are difficult to apply genome-wide; and the widely used MNase-seq suffers from high enzyme cleavage bias and substantial sequencing costs. Developing efficient and accurate computational methods to complement experimental approaches has thus become an important research direction.

Driven by the mechanism of sequence-dependent nucleosome positioning, various computational methods have been developed to predict genome-wide nucleosome positions. Early studies primarily relied on probabilistic models [6, 52, 77, 78]. Segal et al. pioneered a nucleosome positioning model for yeast [6]; Field et al. subsequently refined this model to distinguish nucleosome-forming regions from linker regions [77]; Kaplan et al. extended and improved the model for genome-wide application [52]; Xing et al. also proposed a model discriminating nucleosomal DNA from linker DNA [78]. These methods not only identify nucleosome locations across the genome but also predict their occupancy at specific positions, thereby revealing dynamic regulatory information. Another line of research employs biophysical models, predicting nucleosome positioning by computing the physical and structural properties of DNA [41, 79–89]. Among these, Miele et al. predicted occupancy based on DNA physical attributes [79]; Milani et al. utilized sequence bending energy for occupancy prediction [80]; Locke et al. predicted occupancy by fitting free energy [81]; Van Der Heijden et al. introduced statistical mechanics for occupancy prediction [84]; Wang et al. predicted occupancy based on deformation energy [86]; Liu et al. also used deformation energy for occupancy prediction [89]. Concurrently, machine learning approaches have achieved notable progress in this field [33, 90–95], with most models adopting a binary classification strategy to distinguish nucleosome-forming from nucleosome-inhibiting sequences. Among them, the model proposed by Xi et al. was further extended to directly predict nucleosome occupancy [91]. Although these methods have advanced the field, most still rely on manually extracted sequence features or preset biophysical parameters, limiting their generalizability and automation.

With the rapid development of deep learning techniques [96–103], nucleosome positioning prediction has undergone methodological innovation. Deep learning can automatically learn high-level features from raw DNA sequences, overcoming the limitations of manual feature engineering in traditional methods. In this context, several deep learning-based nucleosome positioning models have emerged [104–109]. These include LeNup by Zhang et al., which combines CNN and GRU [104]; DLNN by Di Gangi et al., integrating CNN and LSTM [105]; CORENup by Amato et al., fusing CNN and LSTM [106]; NP_CBiR by Han et al., combining CNN, GRU, and LSTM [107]; DeepNup by Zhou et al., merging CNN and GRU [108]; and NuPoSe by Masoudi-Sobhanzadeh et al., integrating CNN with ResNet [109]. These models all aim to capture local structural features and long-range contextual dependencies in DNA sequences to improve the discrimination between nucleosomal and linker regions, demonstrating significant performance improvements and application potential.

Despite the encouraging results achieved by existing models, several limitations warrant further exploration. The high cost of obtaining high-resolution experimental data restricts training scale and cross-species generalization for many models. Most models remain confined to a binary classification framework, unable to capture continuous nucleosome occupancy information and thus reflect dynamic regulatory gradients. There is also room for improvement in the architectural design of deep learning models: traditional CNNs have limited receptive fields, making it difficult to model the overall sequence context of the nucleosome core region; stacking layers can easily lead to parameter explosion and gradient instability; while LSTMs can capture long-range dependencies, their bidirectional structures are computationally expensive and difficult to parallelize; residual connections alleviate vanishing gradients, but deep networks and high-dimensional features still introduce high complexity and feature redundancy.

To address these challenges, this study proposes a Hybrid Dilated Gated Separable Convolutional Neural Network (HDGS-Net). Based on convolutional neural networks, it integrates dilated convolution, gated convolution, and depthwise separable convolution, aiming to simultaneously predict nucleosome positioning and occupancy. In this model, dilated convolution flexibly adjusts the dilation rate to expand the receptive field without increasing parameters or network depth, thereby effectively capturing long-range correlations or sequential effects between nucleotides within the nucleosome core region. Gated convolution introduces an adaptive feature selection mechanism, enhancing model expressiveness while mitigating vanishing and exploding gradients. Depthwise separable convolution maintains multi-scale feature extraction capability while effectively reducing computational complexity. The organic combination of these three components enables the model to possess both local perception and global modeling capabilities, while ensuring training stability and optimization robustness. HDGS-Net supports genome-wide, single-base resolution continuous occupancy prediction, providing a richer informational dimension for analyzing genomic regulatory dynamics. Experiments on the benchmark dataset of in vitro genome-wide nucleosome occupancy in Saccharomyces cerevisiae demonstrate that our model outperforms previous methods in its class.

## Materials and methods

### Data source

This study utilized Kaplan et al.‘s [52] in vitro and in vivo nucleosome maps of Saccharomyces cerevisiae (GSE13622), employing only the in vitro data for model training. The in vivo nucleosome map of Schizosaccharomyces pombe [110] was obtained from the GEO database (GSE67410), with its reference genome and associated gene annotations (release 2025-11) downloaded from the PomBase database (https://www.pombase.org/). The reference genomes and corresponding gene annotations for Saccharomyces cerevisiae (SAC2) and Caenorhabditis elegans (WS190), along with the in vivo nucleosome map (adjusted nucleosome coverage) of C. elegans [43], were all retrieved from the UCSC Genome Database (http://genome.ucsc.edu/). Nucleosome occupancy was quantified using the dMean normalization method, defined as the ratio of sequencing reads at each base to the average read count across the whole genome. This approach eliminates sequencing depth variations while providing the model with compliant non-negative input features.

### Stratified screening of nucleosome occupancy sites

**Fig. 1:**
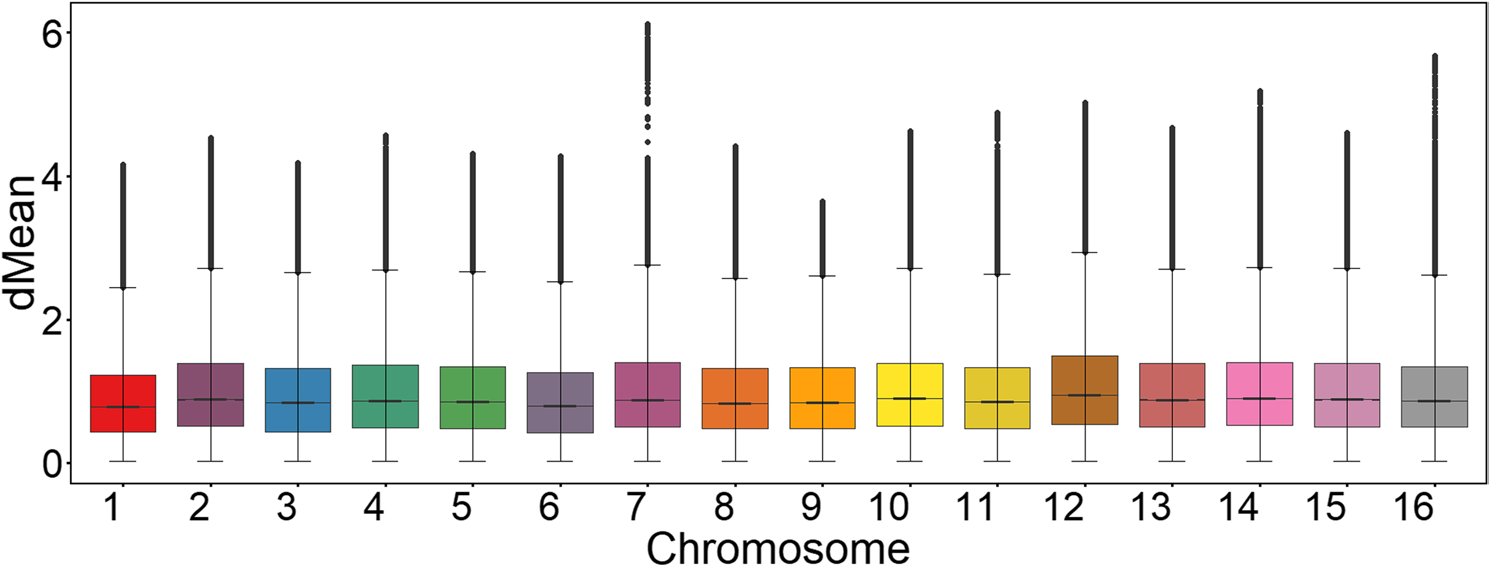
Genome-wide nucleosome occupancy distribution in yeast. Boxplots illustrate the statistical distribution of dMean values for individual chromosomes. The box represents the interquartile range (IQR, Q1–Q3), the midline indicates the median (Q2), and the whiskers extend to Q3 + 1.5 × IQR. Discrete points represent genomic positions with exceptionally high occupancy (dMean ≥ Q3 + 1.5 × IQR), constituting less than 5% of positions per chromosome. The overall data exhibit a conserved left-skewed distribution pattern

We analyzed the genome-wide distribution characteristics of in vitro nucleosome occupancy in Saccharomyces cerevisiae using box plots (Fig. 1). The box represents the interquartile range (IQR = Q3 - Q1), where Q1 and Q3 denote the 25th and 75th percentiles, respectively. The outlier threshold was defined as Q3 + 1.5 × IQR. Analysis revealed a significant left-skewed distribution of dMean values across all chromosomes, characterized by the median (Q2) approaching the lower quartile (Q1). Genomic positions with exceptionally high occupancy (dMean ≥ Q3 + 1.5 × IQR) constituted less than 5% per chromosome. This distribution profile formed the basis for implementing a stratified sampling strategy.

To construct a deep learning model capable of accurately learning nucleosome positioning patterns, we implemented enhanced sampling for regions with strong positioning signals, specifically high nucleosome occupancy regions, during training set construction. High-occupancy regions (> Q3 + 1.5 × IQR) were sampled at a 1:3 ratio, while regions within the normal distribution (≤ Q3 + 1.5 × IQR) were sampled at a 1:30 ratio. During data quality control, we identified anomalies in chromosome 10, potentially stemming from insufficient experimental coverage and technical biases introduced by MNase digestion preference in AT-rich regions. Validation analyses confirmed localized annotation inconsistencies on this chromosome, leading to its complete exclusion from subsequent analyses. Ultimately, we selected 395,314 high-quality sites from the remaining chromosomes to form the training set, while reserving 9,720,129 genomically non-overlapping sites to construct an independent test set (Table S1). This partition scheme, based on mutually exclusive genomic coordinates, fundamentally prevents data leakage between the training and test sets, ensuring a reliable foundation for model performance evaluation.

### Construction of nucleosome sequence feature matrices

**Fig. 2:**
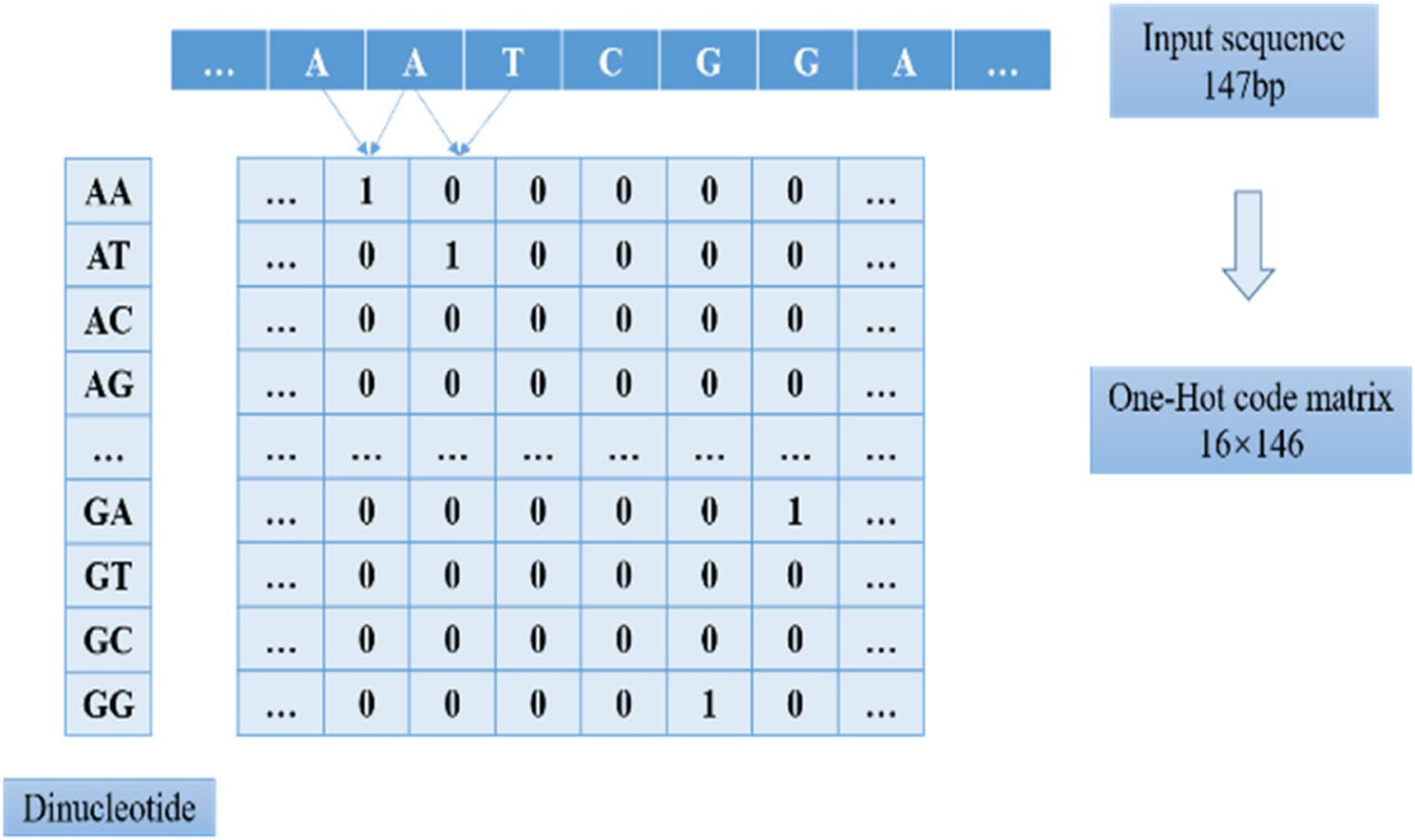
DNA sequence one-hot encoding workflow. A 147 bp sequence is converted into a 16 (feature dimension) × 146 (sequence position) vector matrix through dinucleotide one-hot encoding, with the dMean value of the central site serving as the label. The encoded matrix and its corresponding label are integrated into an HDF5 file

To transform DNA sequences into structured data suitable for convolutional neural network processing, we extracted 73 bp upstream and downstream sequences centered on each genomic site, constructing 147 bp DNA fragments that fully cover the typical binding region of a single nucleosome. We employed a dinucleotide-based one-hot encoding strategy for sequence representation, mapping each consecutive dinucleotide unit in the sequence to a 16-dimensional binary vector where only the dimension corresponding to that specific dinucleotide was assigned a value of 1, with all others set to 0. Consequently, each 147 bp sequence was converted into a feature matrix of dimensions 16 × 146 (Fig. 2). To establish a spatial correspondence between features and labels, we defined the dMean value of the central site of each sequence as the regression target for supervised learning. All 395,314 training samples, comprising their respective feature matrices and labels, were consolidated into an HDF5 format dataset. The test set contained 9,720,129 samples and underwent identical preprocessing to ensure consistent data representation during both training and testing phases.

### Dilated Convolution

Standard convolution expands the receptive field at the cost of a sharp increase in parameters, training instability, or loss of resolution. Its mathematical formulation is as follows:

1 $$(F^\ast k)(x)={\textstyle\sum_\_^{}}\{y\in\Omega\_r\}F(x+y)\cdot k(y)$$

Dilated convolution effectively expands the receptive field without increasing the number of parameters by incorporating a dilation rate parameter. Its mathematical formulation is as follows:

2 $$(F^\ast \_lk)(x)={\textstyle\sum_\_}\{y\in\Omega\_r\}\;F(x+l\cdot y)\cdot k(y)$$

Where *F: Z²* → *R* is the 2D discrete input feature map (nucleosome dinucleotide encoding matrix), *K: Ωr* → *R* is the convolution kernel weight function defined over the neighborhood *Ωr* = {*-r*, ., *r*}², and *l* ∈ *N*⁺ is the dilation rate parameter. This framework was initially proposed by Yu et al. [111]. Chen et al. subsequently proposed an interleaved configuration strategy with different dilation rates to alleviate the grid artifact problem [112]. In the nucleosome occupancy prediction task, by flexibly adjusting the dilation rate l, adaptive capture of input sequence context information can be achieved while maintaining feature resolution, similar to the principle of global feature extraction in pseudo-nucleotide composition [113]. For the one-dimensional sequence characteristics, we adopt a 1×n convolutional kernel architecture, enabling it to progressively integrate local nucleotide patterns along the sequence direction of a single nucleosome into a global representation, thereby preserving local nucleotide combination information while capturing sequential effects or long-range correlations between nucleotides.

### Gated Convolution

Gated convolution regulates the flow of feature information through learnable nonlinear gating units. It is mathematically defined as:

3 $$Y=\left(X^*W+b\right)\cdot\sigma\left(X^*V+c\right)$$

Where *X* ∈ *R*^^(*N*×*m*)^ represents the input feature matrix at layer *l*, *W*,* V* ∈ *R*^^*(k*×*m*×*n)*^ are the feature extraction and gating convolution kernels, respectively, *σ* is the sigmoid activation function, ⊙ denotes element-wise multiplication (Hadamard product), and *b*,* c* ∈ *R*^^*n*^ are bias terms. This mechanism was initially proposed by Dauphin et al. [114] for language modeling tasks, employing gating units to control information retention and forgetting. It helps regulate information flow, preserve critical features, and mitigate vanishing and exploding gradient problems, enabling the network to achieve adaptive feature selection and enhancing model stability and representational capacity.

### Depthwise separable Convolution

Depthwise separable convolution reduces computational burden while maintaining feature extraction capability by decomposing standard convolution into depthwise convolution and pointwise convolution. This method was initially proposed by Chollet [115] and later optimized in several studies [116, 117], gradually becoming a core component of lightweight neural network architectures. Depthwise convolution applies a separate convolutional kernel to each input channel to independently extract spatial features. Its parameter count is:

4 $${Params}_{depthwise}\mathrm{=}{K}^{\mathrm{2}}\cdot{C}_{in}$$

Pointwise convolution uses 1 × 1 convolution to linearly combine the output channels from the depthwise convolution, generating new feature channels. Its parameter count is:

5 $$Params_{pointwise}=C_{in}\cdot{C}_{out}$$

Here, K denotes the convolutional kernel size, while *C*_*in*_ and *C*_*out*_ are the numbers of input and output channels. Compared to the parameter count of standard convolution *K² × C*_*in*_ *× C*_*out*_, the parameter count of depthwise separable convolution *K² × C*_*in*_ *+ C*_*in*_ *× C*_*out*_ is significantly reduced, effectively lowering the model’s computational cost and resource consumption.

### Enhanced convolutional neural network architecture

**Fig. 3:**
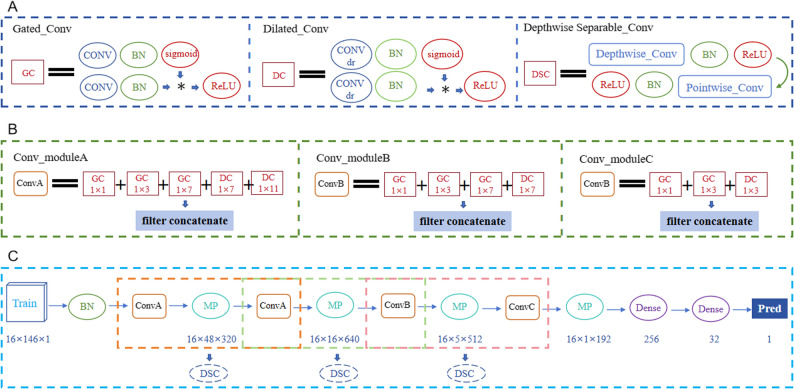
Architecture of the Hybrid Dilated Gated Separable Convolutional Neural Network (HDGS-Net). **A** HDGS-Net integrates the core advantages of Dilated Convolution (DC), Gated Convolution (GC), and Depthwise Separable Convolution (DSC). **B** The network comprises three cascaded parallel convolutional modules (Conv Module A, B, C). Each module incorporates GC and DC branches with varying kernel sizes and dilation rates (dr), interconnected via cascaded DSC units. **C** Feature outputs from each module undergo downsampling through 1 × 3 max-pooling layers prior to being processed by the fully connected network to generate the final prediction value

The Hybrid Dilated Gated Separable Convolutional Neural Network (HDGS-Net, Fig. 3) proposed in this study achieves single-base resolution prediction of nucleosome occupancy across the Saccharomyces cerevisiae genome by systematically integrating the complementary advantages of dilated convolution (DC), gated convolution (GC), and depthwise separable convolution (DSC). The network input is data of size 16 × 146 × 1. After global batch normalization, it sequentially passes through two levels of Conv Module A (a five-branch parallel structure: 1 × 1, 1 × 3, 1 × 7 GC; 1 × 7 DC with dr = 3; and 1 × 11 DC with dr = 5), outputting a feature map of size 16 × 48 × 640. This is followed by Conv Module B (a four-branch structure: 1 × 1, 1 × 3, 1 × 7 GC; 1 × 7 DC with dr = 3), outputting features of size 16 × 5 × 512. Subsequently, Conv Module C (a three-branch structure: 1 × 1, 1 × 3 GC; 1 × 3 DC with dr = 2) outputs features of size 16 × 1 × 192. A 1 × 3 max pooling operation (stride = 3) is applied after each module for downsampling. Between consecutive modules, a cascaded depthwise separable convolution structure is constructed by using the preceding module’s convolution as the depthwise convolution layer and the subsequent module’s convolution as the pointwise convolution layer. All convolutional layers are followed by batch normalization and 40% Dropout to optimize convergence and suppress overfitting. The features output by the final module are flattened and fed into a fully connected network (256→32→1) to generate the predicted value.

### Model training

The model was constructed using the TensorFlow framework and trained with the Adam optimizer. The initial learning rate was set to 1 × 10⁻⁴, with the mean squared error (MSE) serving as the loss function to be minimized, and the mean absolute error (MAE) used for performance evaluation. Training utilized 395,314 samples, split into training, validation, and test sets in a 3:1:1 ratio, with a batch size of 64. The model contains 6.6 million parameters. Training was conducted on an NVIDIA RTX 3090 GPU, with a peak memory usage of 5.6 GB and an average GPU utilization of 53%. The complete training process ran for 200 epochs, taking approximately 20 h in total, with an average duration of 350 s per epoch. During training, callback functions were employed to dynamically adjust the learning rate and automatically save the best-performing model.

## Results

### Prediction of in vitro nucleosome occupancy in Saccharomyces cerevisiae

The HDGS-Net model achieves continuous prediction of in vitro nucleosome occupancy at single-base resolution across the entire Saccharomyces cerevisiae genome. Evaluation on an independent test set demonstrates exceptional robustness, with an average Pearson correlation coefficient of 0.87 between predicted and experimental values across all chromosomes except the anomalous chromosome 10 (Table 1). Chromosome 7 (Chr7) showed the best performance (*R* = 0.89).

**Table 1:** Pearson correlation coefficients between predicted and experimental values of genome-wide in vitro nucleosome occupancy in Saccharomyces cerevisiae

Chr	*R*
Chr1	0.8711
Chr2	0.8294
Chr3	0.8775
Chr4	0.8808
Chr5	0.8784
Chr6	0.8702
Chr7	0.8857
Chr8	0.8740
Chr9	0.8687
Chr10	0.2469
Chr11	0.8758
Chr12	0.8811
Chr13	0.8801
Chr14	0.8785
Chr15	0.8785
Chr16	0.8785

It is noteworthy that our model, along with those of Kaplan et al. [52], Liu et al. [89], and Xi et al. [91], exhibited anomalously low predictive performance on Chr10. This suggests the presence of systematic experimental bias specific to this chromosome, potentially attributable to insufficient local coverage and MNase digestion preference in AT-rich regions [59, 118–121]. Sequence analysis of Chr10 identified systematic deviation within the 120,456–727,158 region. Exclusion of the anomalous 208 bp segment resulted in an improved correlation of 0.78 on chr10. As no Chr10 data were used during model training, this result validates HDGS-Net’s exceptional cross-chromosome predictive capability and generalization performance. Collectively, these findings indicate that the observed anomalies likely originate from localized annotation biases in the experimental data.

**Fig. 4:**
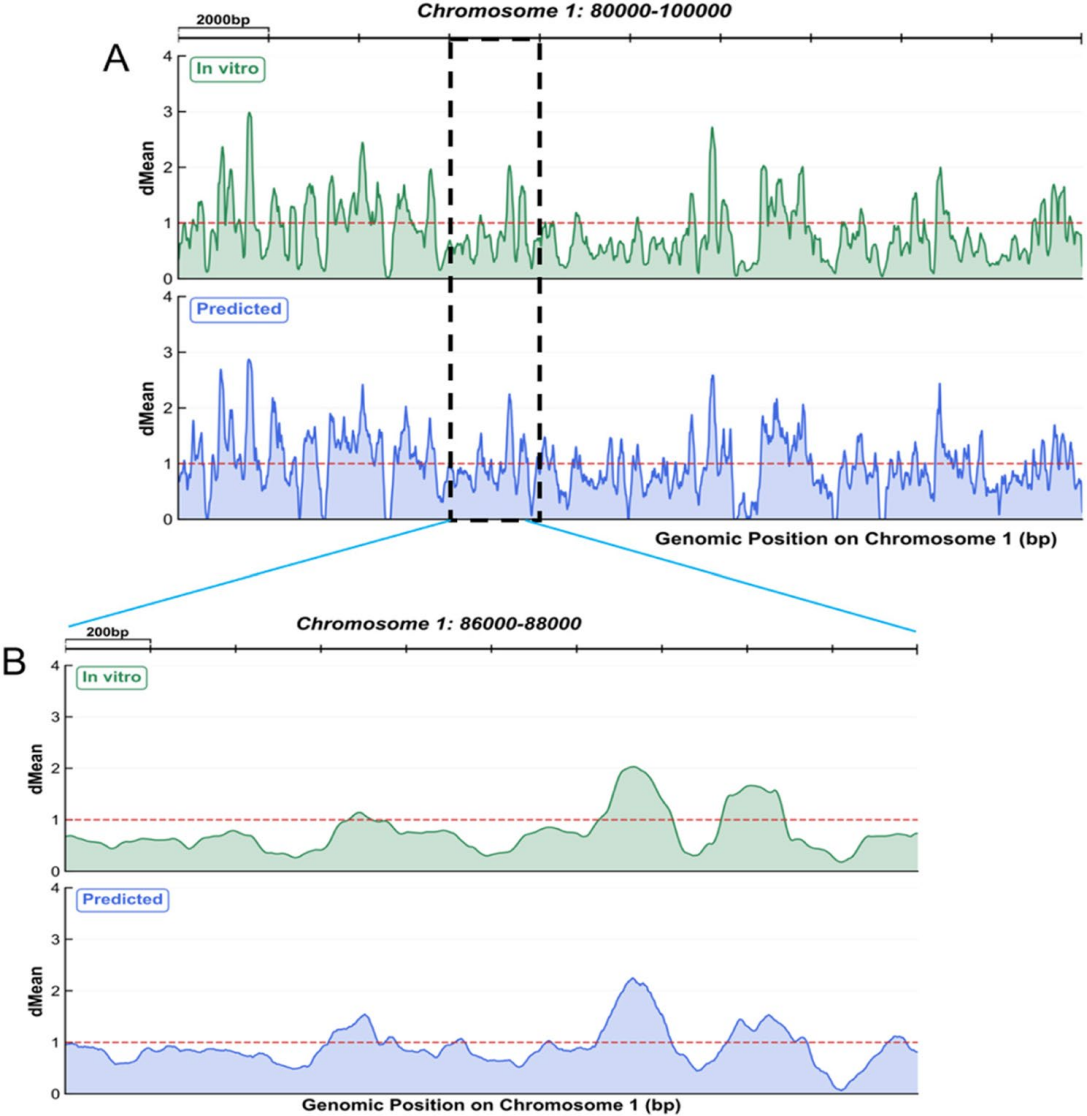
Validation of predicted versus experimental in vitro nucleosome occupancy on Chromosome 1 of Saccharomyces cerevisiae. **A** Macro-scale alignment across the 80–100 kb region demonstrates high consistency between HDGS-Net predictions and experimental observations. The x-axis represents genomic coordinates, while the y-axis indicates dMean values (0–4). **B** Single-base-resolution analysis of the 86–88 kb interval confirms the model’s accurate capture of positioning and occupancy for 10 consecutive nucleosomes. The red dashed line (y = 1) indicates the genome-wide average level

To evaluate the performance of HDGS-Net in predicting in vitro nucleosome occupancy across the whole genome, we conducted a comparative analysis between predicted and experimental values on all 16 chromosomes of Saccharomyces cerevisiae. Figure 4 presents a detailed comparison within the 80–100 kb region of Chromosome 1, with a further zoomed-in view of the 86–88 kb interval showing predictions for 10 consecutive nucleosomes at single-base resolution. This clearly demonstrates HDGS-Net’s capability to capture fine details of nucleosome positioning. Comparative results for the remaining 15 chromosomes are provided in Supplementary Figures S1 to S15.

### Performance comparison with mainstream regression models

**Fig. 5:**
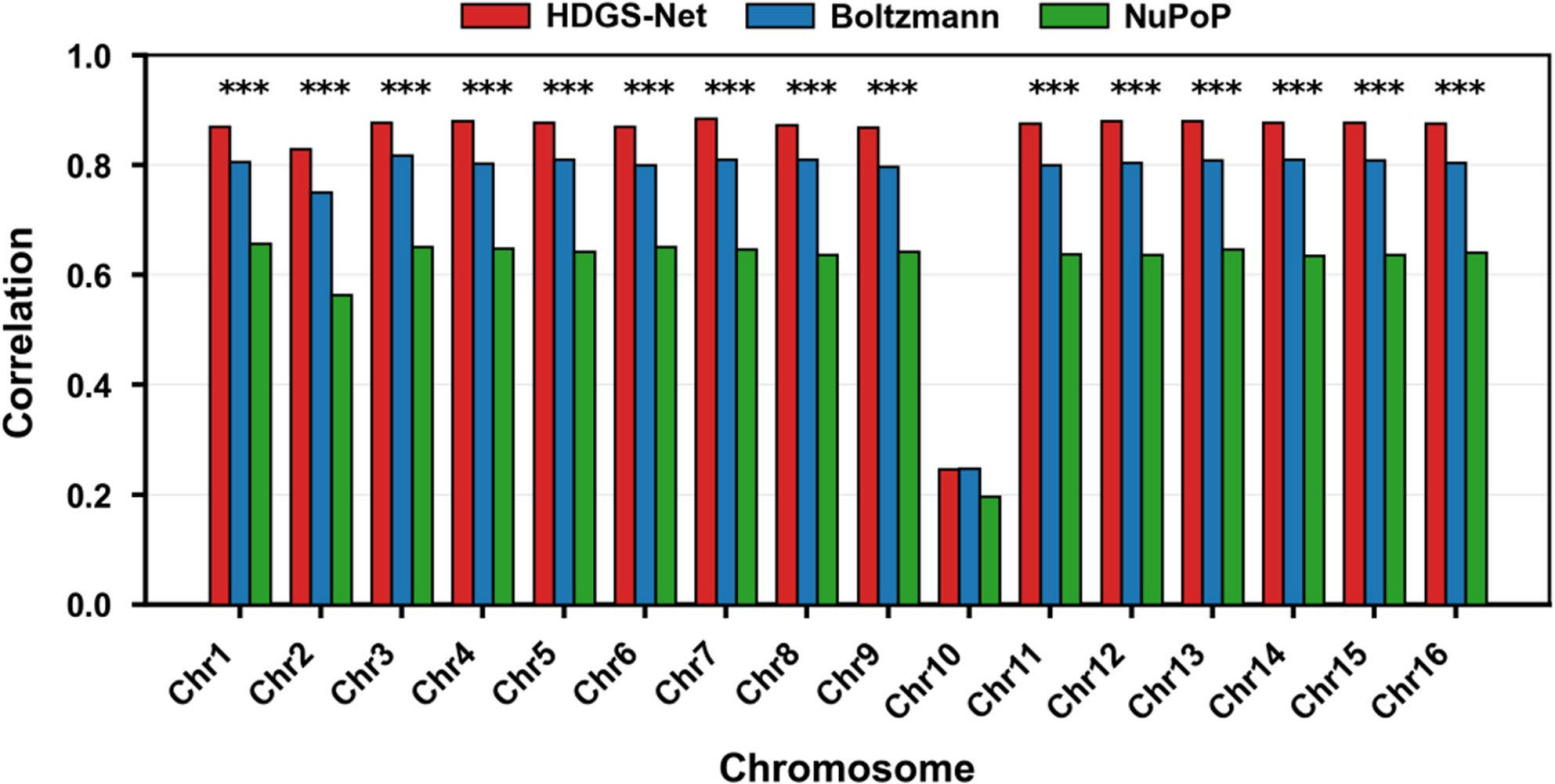
Performance comparison of genome-wide in vitro nucleosome occupancy prediction in Saccharomyces cerevisiae. The distribution of Pearson correlation coefficients across all 16 chromosomes is shown for HDGS-Net (red), the Boltzmann model (blue), and the NuPoP model (green). Except for chromosome 10, HDGS-Net demonstrated superior performance on all other chromosomes, with statistically significant differences compared to the benchmark models (*p* < 0.001)

HDGS-Net demonstrates notable advantages in predicting in vitro nucleosome occupancy. Its genome-wide average correlation coefficient reaches *R* = 0.87, outperforming current mainstream regression models including Kaplan et al. (*R* = 0.84) [52], Liu et al. (Boltzmann model, *R* = 0.80) [89], Xing et al. (*R* = 0.76) [78], Locke et al. (*R* = 0.75) [81], Xi et al. (NuPoP model, *R* = 0.64) [91], and Wang et al. (*R* = 0.47) [86]. It is worth noting that our replication of the Kaplan et al. [52] model yielded a result (*R* = 0.84) that differs from the value reported in their original publication (*R* = 0.89), an observation consistent with that reported by Liu et al. [89]. To further evaluate model performance, we conducted chromosome-wise paired comparisons between HDGS-Net and two benchmark models: the Boltzmann model by Liu et al. [89] and the NuPoP model by Xi et al. [91] (Fig. 5). Detailed predictive values for each chromosome are summarized in Table 2. The results demonstrate that HDGS-Net significantly outperforms both comparative models (*p* < 0.001) on all chromosomes except chromosome 10, fully validating its more robust and superior predictive capability for nucleosome occupancy across the entire genome.

**Table 2:** Performance comparison of genome-wide in vitro nucleosome occupancy prediction models in Saccharomyces cerevisiae

Yeast	HGDS-Net	Boltzmann	NuPoP
Chr1	0.8711	0.806	0.657
Chr2	0.8294	0.751	0.564
Chr3	0.8775	0.818	0.652
Chr4	0.8808	0.803	0.649
Chr5	0.8784	0.811	0.643
Chr6	0.8702	0.801	0.652
Chr7	0.8857	0.810	0.647
Chr8	0.874	0.811	0.636
Chr9	0.8687	0.798	0.642
Chr10	0.2469	0.248	0.197
Chr11	0.8758	0.801	0.638
Chr12	0.8811	0.805	0.637
Chr13	0.8801	0.809	0.647
Chr14	0.8785	0.810	0.635
Chr15	0.8785	0.809	0.637
Chr16	0.8771	0.805	0.641

### Performance comparison with machine learning models

To comprehensively evaluate the predictive performance of HDGS-Net, we used chromosome 1 as the training set and compared it with two mainstream deep learning architectures (LeNup, DeepNup) [104, 108] and two traditional machine learning models (Random Forest, XGBoost). To adapt to the regression task of nucleosome occupancy, we modified the originally classification-designed deep learning models by retaining their backbone architectures, changing the output layer activation function to ReLU, and setting the loss function to mean squared error (MSE). All models were trained on a unified dataset (60% training, 20% validation, 20% test) using mean absolute error (MAE) and coefficient of determination (R²) as evaluation metrics.

**Fig. 6:**
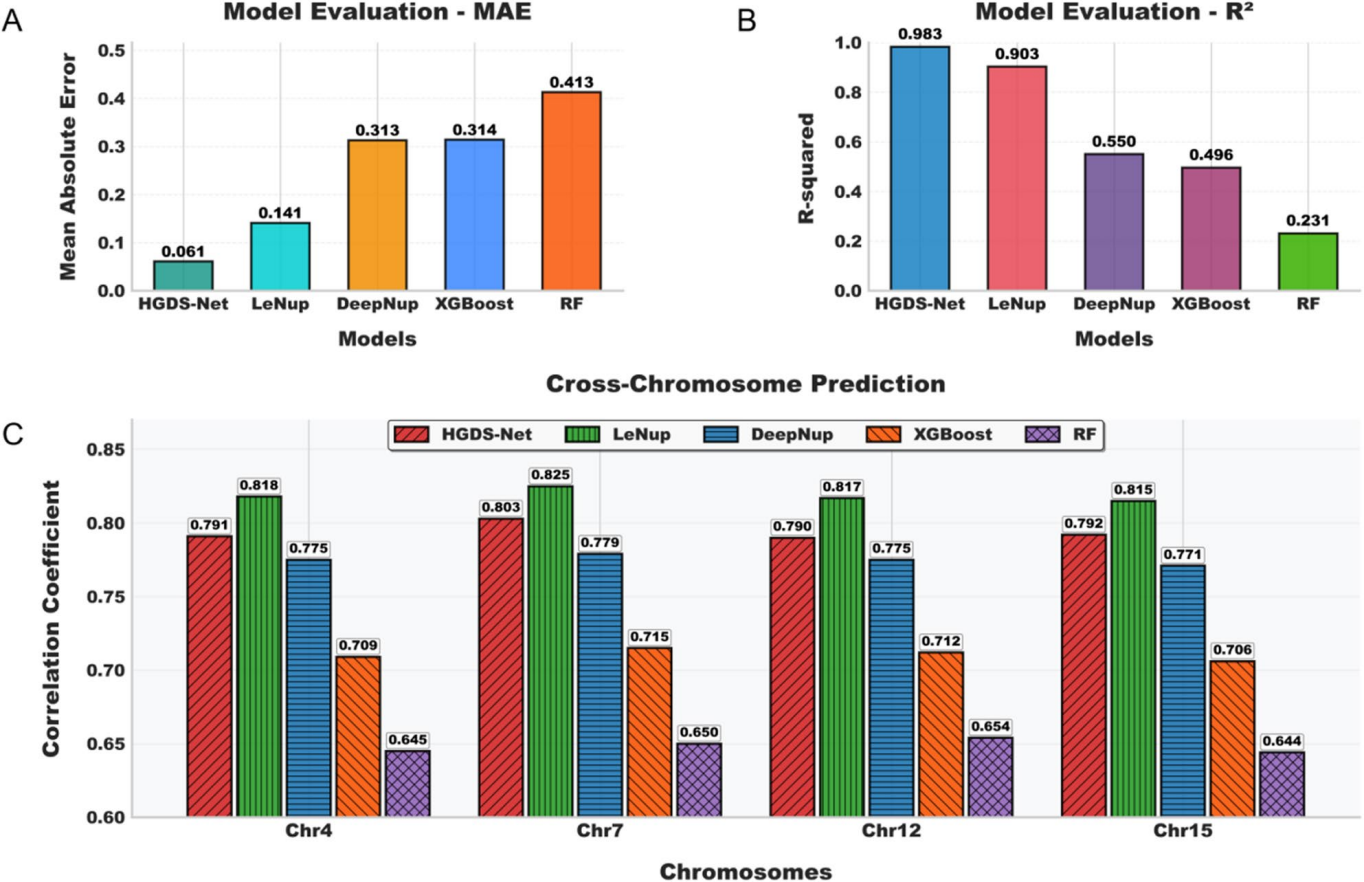
Performance comparison of HDGS-Net with multiple models. **A** On the training chromosome (Chr1), HDGS-Net demonstrates lower mean absolute error (MAE) than both traditional machine learning and deep learning models. **B** On the same training chromosome (Chr1), HDGS-Net also achieves higher coefficient of determination (R²) compared to other models. **C** Evaluation of average generalization performance across four unseen chromosomes (Chr4, 7, 12, 15). The results indicate that HDGS-Net not only achieves optimal performance on training data but also demonstrates exceptional robust cross-chromosome generalization capability in independent testing

The experimental results demonstrate that HDGS-Net’s performance on chromosome 1 (MAE = 0.06, R²=0.98) indicates its superiority in both prediction accuracy and fitting capability over the other six models (Figs. 6A-B). To further evaluate generalization ability, we applied the trained models to four unseen chromosomes (Chr4, Chr7, Chr12, Chr15). HDGS-Net achieved an average Pearson correlation coefficient (*R* = 0.794) on these four chromosomes, ranking second (Fig. 6C), slightly below the structurally adapted LeNup model (*R* = 0.819) but superior to all traditional machine learning methods (Fig. 6C). Notably, all four deep learning models outperformed traditional machine learning models in cross-chromosome prediction.

Although the deep learning models included in the comparison were originally designed for classification tasks, they demonstrated competent regression prediction capability after adaptation, reflecting the effectiveness of our modifications. More importantly, HDGS-Net exhibited consistent and robust superior performance on unseen chromosomes, achieving optimal comprehensive evaluation metrics among these models. This indicates its ability to effectively capture universal patterns between sequences and nucleosome occupancy without overfitting to the training data, demonstrating exceptional cross-chromosome generalization capability and genome-wide prediction potential.

### Validation of sequence determinants of nucleosome occupancy

**Fig. 7:**
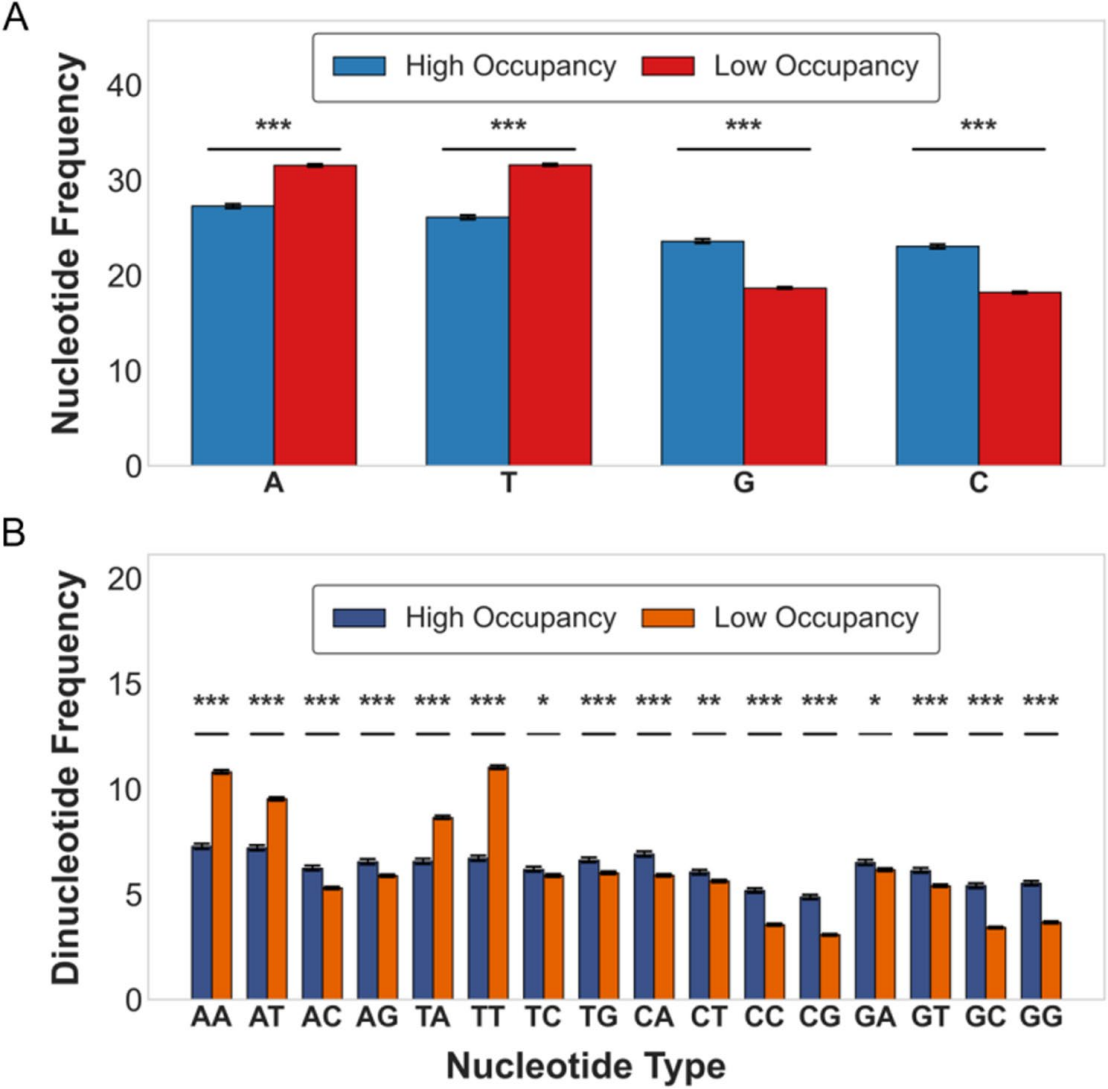
Nucleotide composition characteristics of different occupancy groups. **A** Mononucleotide distribution. The high occupancy group shows enrichment of G and C, while the low occupancy group shows enrichment of A and T. **B** Dinucleotide distribution. GG and CC are significantly enriched in the high occupancy group, while AA and TT are significantly enriched in the low occupancy group

We performed nucleotide composition analysis on the Chromosome 1 test set of Saccharomyces cerevisiae (Fig. 7). Based on predicted in vitro nucleosome occupancy, sequences were divided into high and low occupancy groups using the quartile method. Mononucleotide analysis revealed that A and T content in the high occupancy group was lower than in the low occupancy group, while G and C content was higher in the high occupancy group (Fig. 7A). Dinucleotide analysis showed significant enrichment of GG and CC in the high occupancy group (*p* < 0.001), and significant enrichment of AA and TT in the low occupancy group (Fig. 7B). These results indicate that nucleosomes tend to bind regions enriched with GG and CC, while excluding regions rich in AA and TT [75, 122–126].

**Fig. 8:**
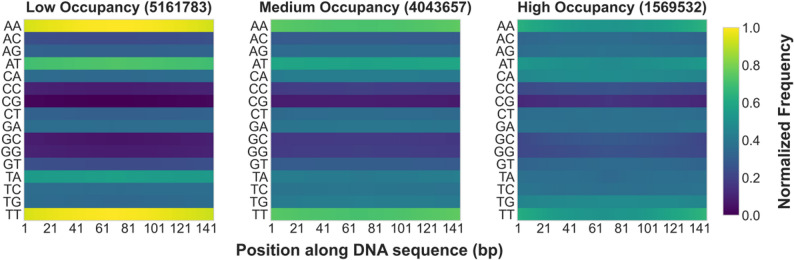
Association analysis between in vitro nucleosome occupancy and dinucleotide frequencies in Saccharomyces cerevisiae. The heatmap displays normalized frequency distributions of different dinucleotides across 147 bp DNA sequences, with dinucleotide types on the vertical axis and nucleotide positions on the horizontal axis. AT-type dinucleotides are enriched in low-occupancy regions, while GC-type dinucleotides are enriched in high-occupancy regions

For further validation of the aforementioned patterns, we generated a digital matrix through dinucleotide one-hot encoding of complete genomic sequences and classified them into high, medium, and low occupancy groups using K-means clustering based on nucleosome occupancy levels (Figure S16). Heatmap analysis demonstrated that AT-type dinucleotide frequencies decrease with rising occupancy, whereas CG-type dinucleotide frequencies increase correspondingly (Fig. 8). Subsequent analysis involved dividing the digital matrix into ten equal groups after sorting by occupancy in descending order. This revealed enrichment of AT-type dinucleotides in the lowest occupancy group (G10, Figure S17), and enrichment of GC-type dinucleotides in the highest occupancy group (G1), with GC dinucleotide frequency consistently exceeding other dinucleotides within its category (Figure S18). Among mixed dinucleotides, CA displayed relatively high frequency while AC showed the lowest frequency (Figure S19). In transition dinucleotides, TG frequency remained relatively low (Figure S20). These analyses collectively confirm that nucleosome occupancy is regulated by DNA sequence characteristics: GC-rich sequences facilitate stable nucleosome binding, while AT-rich sequences inhibit nucleosome formation [20, 69, 71, 124, 127–133].

### Analysis of nucleosome sequence features in transcription start site regions

To decipher the sequence features of nucleosomes in transcription start site (TSS) regions of Saccharomyces cerevisiae, we categorized them into two classes: those with a nucleosome-free region (SNFR) and those without (NNFR). Each 147 bp sequence was encoded as a 16 × 146 matrix and classified using the HDGS-Net model, with a sigmoid activation function in the output layer. Model performance was evaluated via five-fold cross-validation.

Initial classification revealed no significant differences in accuracy between SNFR and NNFR on either the forward or reverse strands across six nucleosome positions (Tables S2–S3). Therefore, data from both strands were merged. Further distinction between SNFR and NNFR did not improve classification performance (Table S4), indicating highly similar sequence patterns between the two categories and supporting their integration into a unified dataset. Classification of symmetric nucleosome positions (–1 and + 1) also showed no significant effect (Table S5). Subsequent analysis was conducted based on nucleosome position groups (1NFR, 2NFR, 3NFR). Pairwise classification among the three groups likewise revealed no significant discriminability (Table S6).

**Fig. 9:**
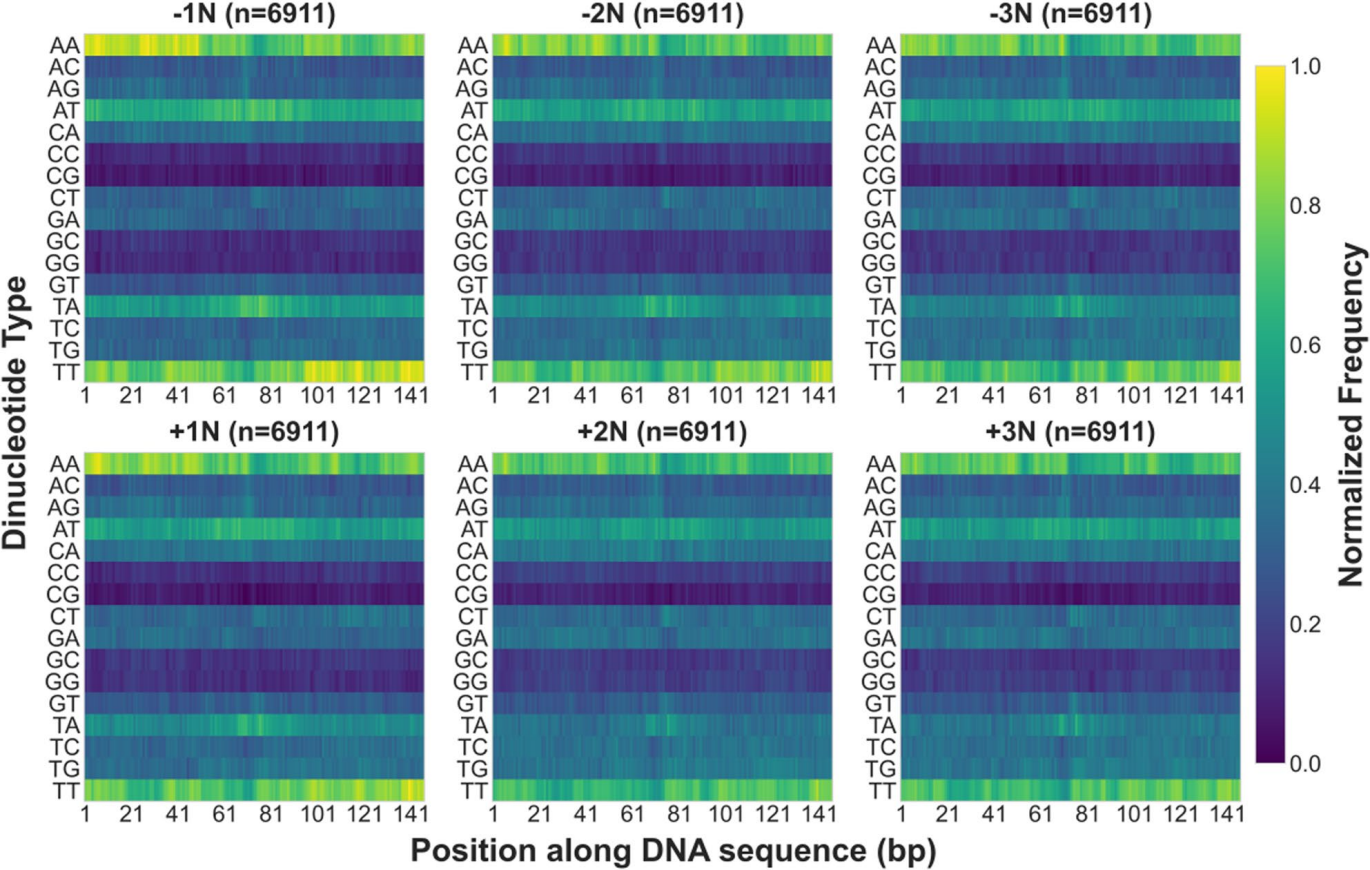
Heatmap of sequence features of symmetric nucleosomes in transcription start site regions. The high sequence similarity between symmetric nucleosomes validates the dominant role of intrinsic DNA sequence preference in nucleosome positioning

Heatmap analysis further confirmed that SNFR and NNFR exhibit highly consistent sequence features at identical positions on both DNA strands (Figures S21–S22). Symmetric nucleosomes displayed strongly conserved sequence patterns (Fig. 9), with minimal inter-group variation between symmetric pairs (Figure S23). These results demonstrate that nucleosome sequence characteristics in the TSS region are highly conserved, irrespective of the presence of a nucleosome-free region, thereby validating the dominant role of intrinsic sequence preferences in nucleosome positioning [6, 52–55].

### Sequence dependence of nucleosome positioning across species

To assess the intrinsic guiding role of DNA sequence in nucleosome positioning, we constructed datasets containing 972, 5,027, and 2,786 transcription start sites for Schizosaccharomyces pombe, Saccharomyces cerevisiae, and Caenorhabditis elegans, respectively, with the nematode data representing a randomly selected 20% sample. These TSS sites were matched with in vivo nucleosome occupancy data. Using each TSS as the center, we precisely extracted positions at 10 bp intervals within the upstream and downstream 1000 bp regions along with their corresponding occupancy values, while recording strand orientation. Each position was then centered to extract 73 bp upstream and downstream, constructing 147 bp sequence fragments. For positions on the negative strand, sequences were converted to their complementary sequences. The label for each sequence was defined as the nucleosome occupancy value at its central position, with all in vivo occupancy data normalized using the dMean method.

Using the HDGS-Net model trained on S. cerevisiae in vitro data to predict nucleosome occupancy across the three species, results showed the highest average correlation between predicted and experimental values in C. elegans (*R* = 0.61), followed by S. cerevisiae (*R* = 0.57), with S. pombe being the lowest (*R* = 0.29). These results are highly consistent with known nucleosome positioning characteristics of each species. S. pombe exhibits positive correlation between AT content and nucleosome occupancy, along with absence of distinct nucleosome-depleted regions [60], contrasting sharply with S. cerevisiae’s preference for GC sequences, suggesting its nucleosome positioning is primarily regulated by non-sequence factors such as chromatin remodeling complexes. In contrast, the high prediction accuracy for C. elegans confirms the dominant role of DNA sequence in its nucleosome assembly, with potential mechanisms including bidirectional promoter structures requiring specific nucleosome arrangements [134], AT-rich sequences preset transcriptional response potential through nucleosome instability [135], and close associations between specific DNA structural parameters and nucleosome occupancy [136]. This study quantifies the differential contributions of DNA sequence to nucleosome positioning across three eukaryotic species through computational modeling, confirming sequentially decreasing quantitative contributions in C. elegans, S. cerevisiae, and S. pombe, providing new perspectives for understanding evolutionary mechanisms of chromatin organization.

## Discussion

HDGS-Net demonstrates exceptional performance in predicting genome-wide in vitro nucleosome occupancy in Saccharomyces cerevisiae, achieving higher predictive accuracy than traditional computational methods while maintaining competitive advantages when compared with adaptively modified deep learning models. These results indicate that in this model organism, the intrinsic properties of DNA sequences play a decisive role in nucleosome positioning and occupancy levels. The model’s success derives from the synergistic effects of its innovative architecture: dilated convolution effectively captures long-range sequence patterns across nucleosome regions, gated convolution enables adaptive feature selection, and depthwise separable convolution preserves multi-scale feature extraction capability. This multi-component collaborative working mechanism empowers the model to autonomously discover discriminative features directly from raw sequence data.

Sequence analysis further validates the deterministic principles of nucleosome occupancy. AA or TT dinucleotides enriched in low-occupancy regions inhibit nucleosome binding, whereas GC or CG dinucleotides enriched in high-occupancy regions promote stable nucleosome binding. These computational results are consistent with in vitro chromatin reconstitution experiments, providing computational evidence that intrinsic DNA physical properties play a central role in nucleosome positioning. Notably, analysis of TSS regions demonstrates that even under different chromatin environments, the sequence features of flanking nucleosomes remain highly conserved, with nearly indistinguishable sequence patterns at symmetric positions, further confirming the universal dominant role of sequence preference. Cross-species analysis verifies that the guiding efficacy of DNA sequences in nucleosome positioning varies among species, showing progressively decreasing quantitative contributions in Caenorhabditis elegans, Saccharomyces cerevisiae, and Schizosaccharomyces pombe. Regarding the anomalous behavior of chromosome 10, multiple models consistently exhibited systematic bias, suggesting potential digestion preference of MNase-seq technology in high AT-content regions. After excluding specific anomalous 208 bp segments, HDGS-Net’s predictive performance immediately recovered to normal levels, which not only reveals potential data quality issues but also corroborates the model’s inherent robustness. Cross-chromosome testing further demonstrates that HDGS-Net captures universal sequence patterns rather than simply overfitting the training data.

Notwithstanding the significant advances achieved by HDGS-Net, we must acknowledge its current limitations. The present model, trained exclusively on in vitro reconstitution data, effectively deciphers intrinsic sequence-encoded preferences but does not encompass the complex epigenetic regulatory networks operative in vivo. Future research will focus on developing unified predictive frameworks that integrate in vivo data from multiple species. Building upon sequence determinism, this work aims to systematically elucidate the multi-level, multi-dimensional regulatory relationships between dynamic higher-order chromatin assembly and precise nucleosome positioning.

## Conclusion

The deep learning framework HDGS-Net developed in this study enables continuous prediction of in vitro nucleosome occupancy at single-base resolution across the entire Saccharomyces cerevisiae genome. By innovatively integrating dilated convolution, gated convolution, and depthwise separable convolution, the model effectively overcomes the receptive field limitations and feature extraction bottlenecks inherent in traditional prediction methods, achieving improvements in both prediction accuracy and generalization capability. Sequence feature analysis confirms that the physical properties of DNA dinucleotides play a decisive role in nucleosome occupancy, where AT-rich sequences inhibit nucleosome binding while GC-rich sequences promote stable nucleosome binding. Analysis of transcription start regions demonstrates that regardless of the presence of nucleosome-free regions, flanking nucleosomes exhibit highly conserved sequence features, supporting the universal regulatory role of sequence preference in nucleosome positioning. Cross-species analysis verifies species-specific differences in the guiding efficacy of DNA sequences on nucleosome positioning, with progressively decreasing quantitative contributions observed in Caenorhabditis elegans, Saccharomyces cerevisiae, and Schizosaccharomyces pombe, providing new perspectives for understanding chromatin organization mechanisms across species. This study offers a high-accuracy predictive tool for investigating dynamic nucleosome positioning.

## Supplementary Information

Supplementary Material 1.

## Data Availability

The demonstration datasets, core algorithm code, reproducible result files, and graphical resources from this study are permanently archived in the GitHub repository and accessible via: https://github.com/SFQ77/HDGS-Net.
