# Supplementary material for: HDGS-Net: nucleosome occupancy prediction based on a hybrid dilated gated separable convolutional neural network
Source: BMC Genomics. 2026 Jan 24;27:209. doi: 10.1186/s12864-026-12523-2 (PMC12910957; doi:10.1186/s12864-026-12523-2)
Supplement: Supplementary file 1 — Supplementary Material 1. [file 12864_2026_12523_MOESM1_ESM.docx]

**Supplementary material**

****Table S1.**** Screening Statistics of in vitro Nucleosome Occupancy Sites Across Chromosomes in the Saccharomyces cerevisiae Genome.

| Chromosome | Primordial Site | Deep Learning Site | Test Site |
| --- | --- | --- | --- |
| Chr1 | 168234 | 6557 | 161677 |
| Chr2 | 748431 | 29077 | 719354 |
| Chr3 | 244966 | 9140 | 235826 |
| Chr4 | 1378420 | 54086 | 1324334 |
| Chr5 | 515003 | 19920 | 495083 |
| Chr6 | 236402 | 8793 | 227609 |
| Chr7 | 983305 | 38445 | 944860 |
| Chr8 | 484641 | 19040 | 465601 |
| Chr9 | 382415 | 14270 | 368145 |
| Chr10 | 659529 | 25671 | 633858 |
| Chr11 | 632626 | 25094 | 607532 |
| Chr12 | 931931 | 36380 | 895551 |
| Chr13 | 848262 | 33587 | 814675 |
| Chr14 | 706446 | 27752 | 678694 |
| Chr15 | 997401 | 38604 | 958797 |
| Chr16 | 856960 | 34569 | 822391 |
| Total | 10774972 | 420985(3.91%) | 10353987 |
| Total_without_10 | 10115443 | 395314 (3.67%) | 9720129 |

****Table S2.** Binary Classification Results of Corresponding Nucleosomes on Positive and Negative Strands in SNFR.**

| ±SNFR | Sample | Accuracy | Precision | Recall | F1-score |
| --- | --- | --- | --- | --- | --- |
| -1 | 4542 | 0.5892 | 0.5981 | 0.5847 | 0.5885 |
| -2 | 4542 | 0.5115 | 0.5174 | 0.5169 | 0.5066 |
| -3 | 4542 | 0.4988 | 0.5095 | 0.3892 | 0.4224 |
| +1 | 4542 | 0.7151 | 0.7469 | 0.6733 | 0.7021 |
| +2 | 4542 | 0.6493 | 0.6760 | 0.6013 | 0.6294 |
| +3 | 4542 | 0.6209 | 0.6344 | 0.6196 | 0.6189 |

****Table S3.** Binary Classification Results of Corresponding Nucleosomes on Positive and Negative Strands in NNFR.**

| ±NNFR | Sample | Accuracy | Precision | Recall | F1-score |
| --- | --- | --- | --- | --- | --- |
| -1 | 2369 | 0.4914 | 0.4841 | 0.9229 | 0.6359 |
| -2 | 2369 | 0.4884 | 0.3811 | 0.7840 | 0.5127 |
| -3 | 2369 | 0.5108 | 0.4100 | 0.4901 | 0.3806 |
| +1 | 2369 | 0.5078 | 0.1910 | 0.3956 | 0.2576 |
| +2 | 2369 | 0.5048 | 0.1897 | 0.4000 | 0.2573 |
| +3 | 2369 | 0.4994 | 0.6078 | 0.6213 | 0.4242 |

****Table S4.** Binary Classification Results of Corresponding Nucleosomes in SNFR and NNFR.**

| SNFR - NNFR | Sample | Accuracy | Precision | Recall | F1-score |
| --- | --- | --- | --- | --- | --- |
| -1 | 6911 | 0.5944 | 0.4127 | 0.4238 | 0.4119 |
| -2 | 6911 | 0.5990 | 0.3743 | 0.2722 | 0.3009 |
| -3 | 6911 | 0.6003 | 0.3525 | 0.1946 | 0.2432 |
| +1 | 6911 | 0.5761 | 0.3602 | 0.2975 | 0.2976 |
| +2 | 6911 | 0.5625 | 0.3380 | 0.3031 | 0.2992 |
| +3 | 6911 | 0.5863 | 0.3341 | 0.2123 | 0.2548 |

****Table S5.** Binary Classification Results of Symmetric Nucleosomes in TSS Regions.**

| TSS | Sample | Accuracy | Precision | Recall | F1-score |
| --- | --- | --- | --- | --- | --- |
| 1 | 13822 | 0.5679 | 0.5758 | 0.5325 | 0.5461 |
| 2 | 13822 | 0.5838 | 0.5876 | 0.5634 | 0.5742 |
| 3 | 13822 | 0.5232 | 0.5217 | 0.5553 | 0.5281 |

****Table S6.** Binary Classification Results of Nucleosomes in Symmetric Groups in TSS Regions.**

| TSS | Sample | Accuracy | Precision | Recall | F1-score |
| --- | --- | --- | --- | --- | --- |
| 1-2 | 27644 | 0.6113 | 0.6266 | 0.5681 | 0.5892 |
| 1-3 | 27644 | 0.6370 | 0.6341 | 0.6569 | 0.6430 |
| 2-3 | 27644 | 0.4997 | 0.4946 | 0.5687 | 0.4785 |


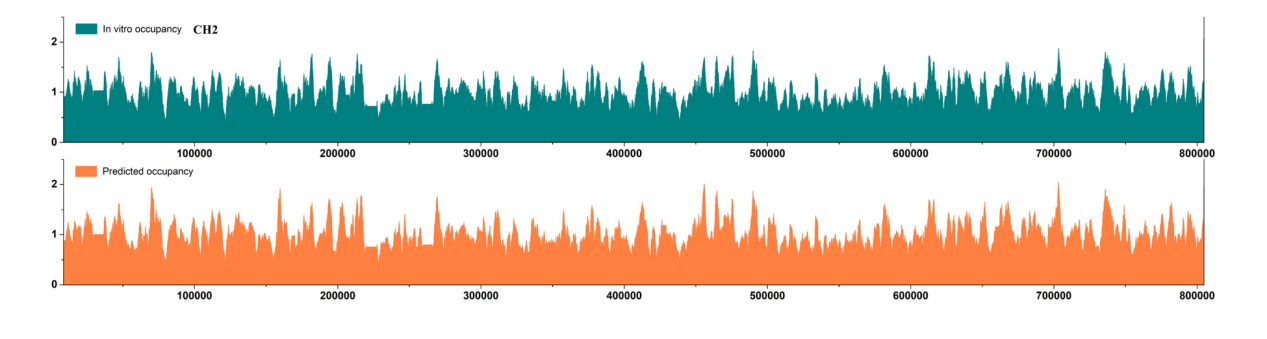
**Figure S1. Comparison of experimental observations and model predictions of in vitro nucleosome occupancy on Saccharomyces cerevisiae chromosome 2. The experimental observation curve (green) and HDGS-Net prediction curve (orange) show high consistency within the 0-800,000 bp region. The x-axis represents chromosomal position, and the y-axis represents nucleosome occupancy.**


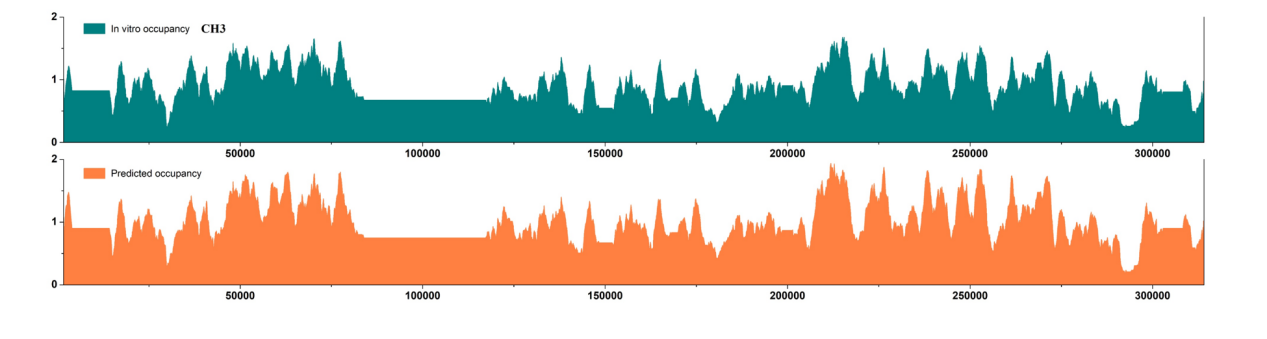


**Figure S2. Comparison of experimental observations and model predictions of in vitro nucleosome occupancy on Saccharomyces cerevisiae chromosome 3. The experimental observation curve (green) and HDGS-Net prediction curve (orange) show high consistency within the 0-300,000 bp region. The x-axis represents chromosomal position, and the y-axis represents nucleosome occupancy.**


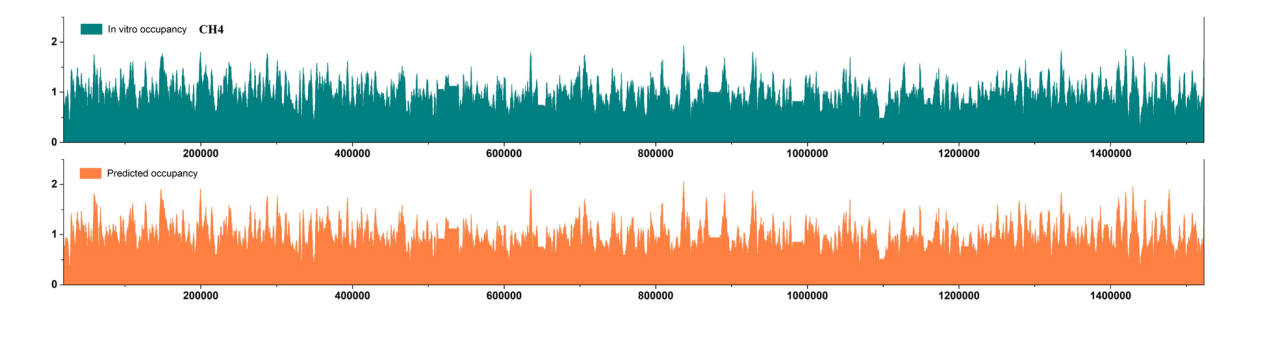


**Figure S3. Comparison of experimental observations and model predictions of in vitro nucleosome occupancy on Saccharomyces cerevisiae chromosome 4. The experimental observation curve (green) and HDGS-Net prediction curve (orange) show high consistency within the 0-1,400,000 bp region. The x-axis represents chromosomal position, and the y-axis represents nucleosome occupancy.**


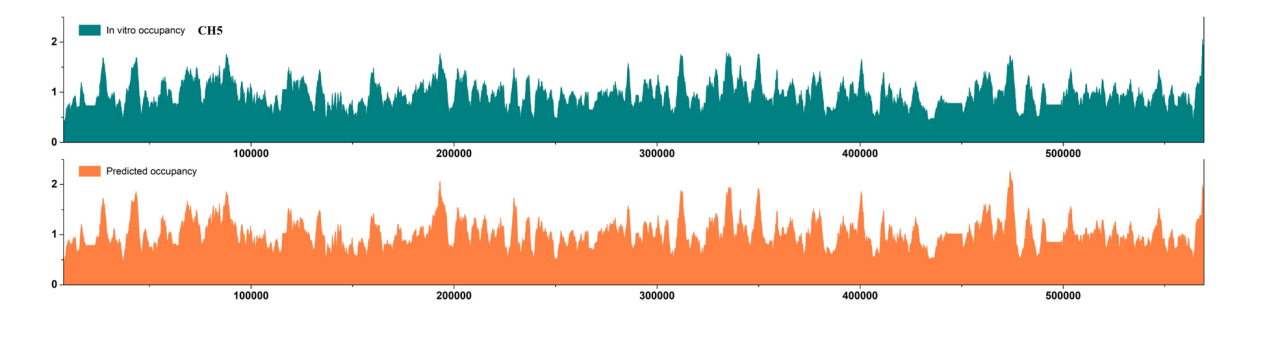


**Figure S4. Comparison of experimental observations and model predictions of in vitro nucleosome occupancy on Saccharomyces cerevisiae chromosome 5. The experimental observation curve (green) and HDGS-Net prediction curve (orange) show high consistency within the 0-500,000 bp region. The x-axis represents chromosomal position, and the y-axis represents nucleosome occupancy.**


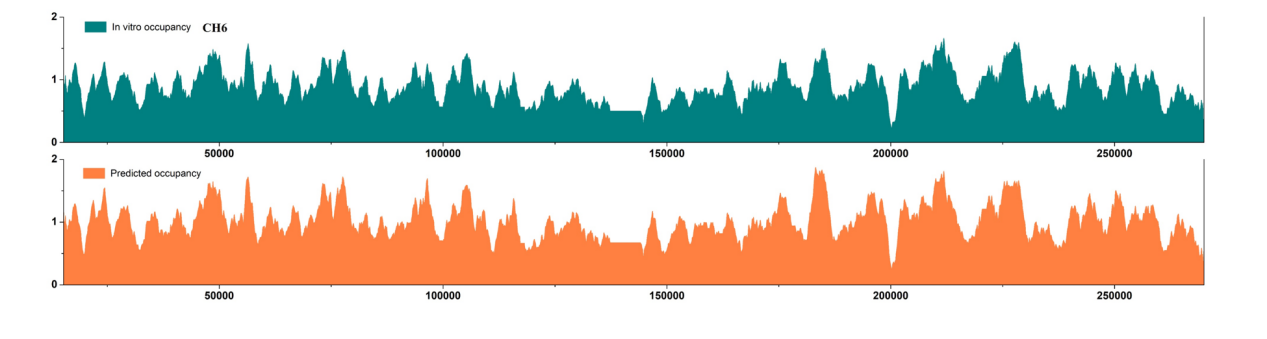


**Figure S5. Comparison of experimental observations and model predictions of in vitro nucleosome occupancy on Saccharomyces cerevisiae chromosome 6. The experimental observation curve (green) and HDGS-Net prediction curve (orange) show high consistency within the 0-250,000 bp region. The x-axis represents chromosomal position, and the y-axis represents nucleosome occupancy.**


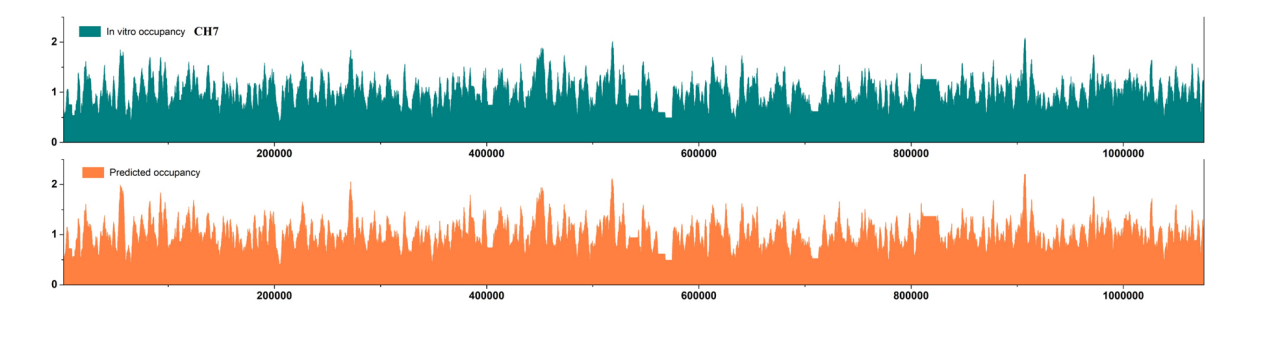


**Figure S6. Comparison of experimental observations and model predictions of in vitro nucleosome occupancy on Saccharomyces cerevisiae chromosome 7. The experimental observation curve (green) and HDGS-Net prediction curve (orange) show high consistency within the 0-1,000,000 bp region. The x-axis represents chromosomal position, and the y-axis represents nucleosome occupancy.**


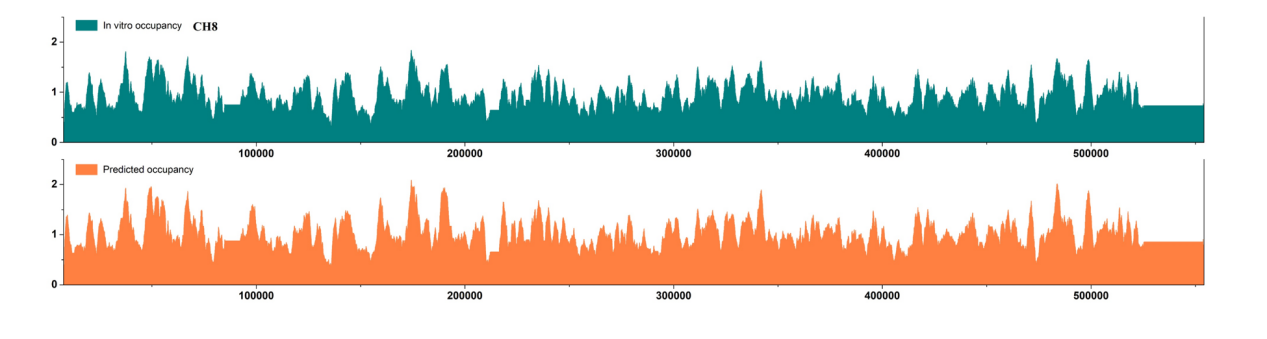


**Figure S7. Comparison of experimental observations and model predictions of in vitro nucleosome occupancy on Saccharomyces cerevisiae chromosome 8. The experimental observation curve (green) and HDGS-Net prediction curve (orange) show high consistency within the 0-500,000 bp region. The x-axis represents chromosomal position, and the y-axis represents nucleosome occupancy.**


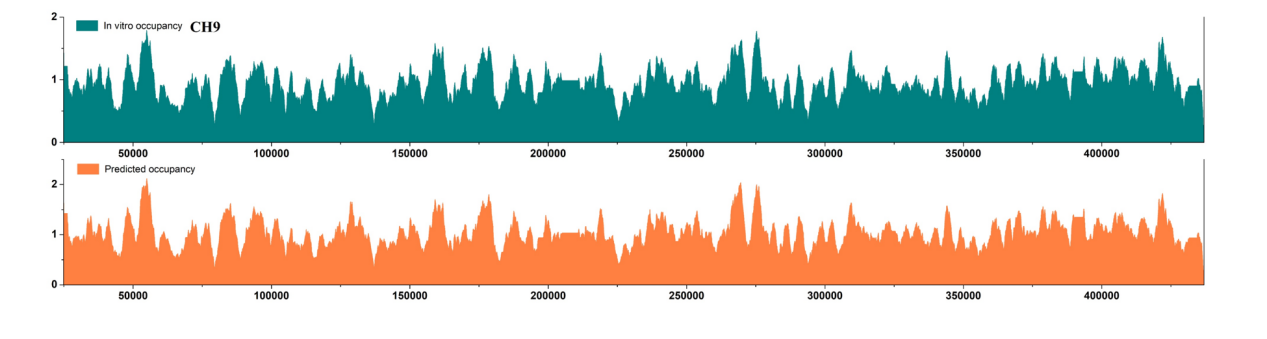


**Figure S8. Comparison of experimental observations and model predictions of in vitro nucleosome occupancy on Saccharomyces cerevisiae chromosome 9. The experimental observation curve (green) and HDGS-Net prediction curve (orange) show high consistency within the 0-400,000 bp region. The x-axis represents chromosomal position, and the y-axis represents nucleosome occupancy.**


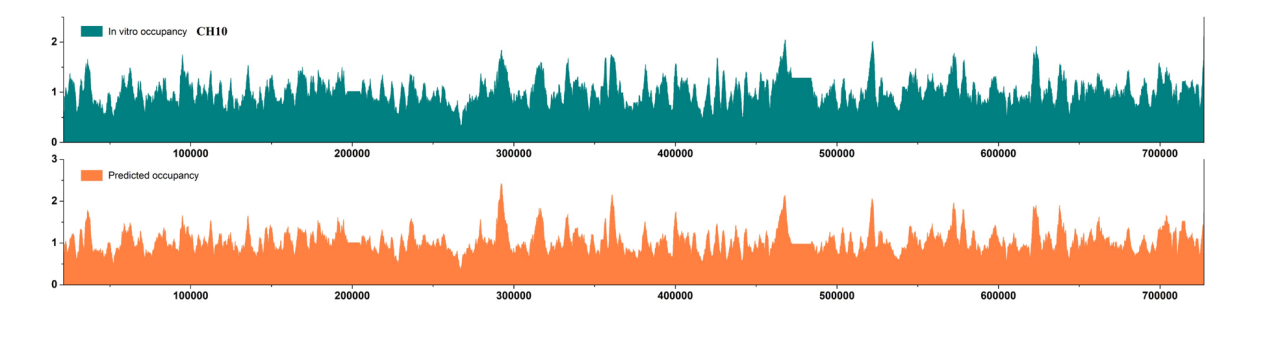


**Figure S9. Comparison of experimental observations and model predictions of in vitro nucleosome occupancy on Saccharomyces cerevisiae chromosome 10. The experimental observation curve (green) and HDGS-Net prediction curve (orange) show high consistency within the 0-700,000 bp region. The x-axis represents chromosomal position, and the y-axis represents nucleosome occupancy.**


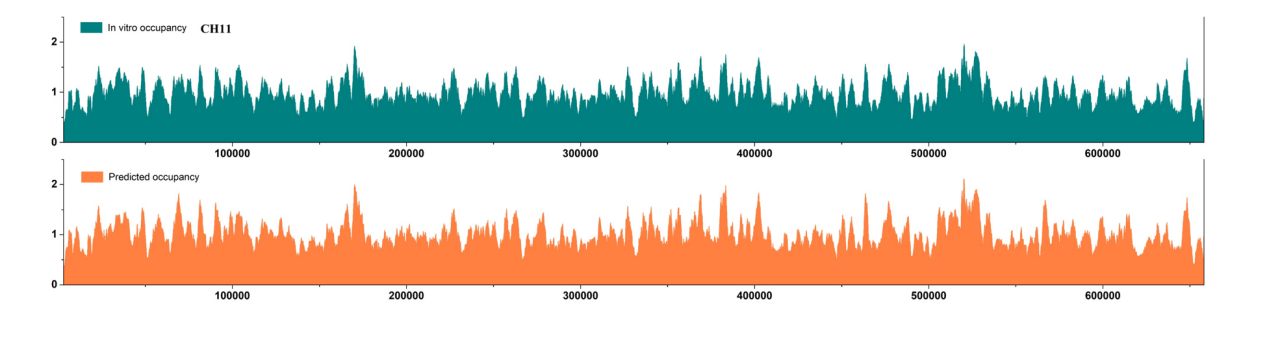


**Figure S10. Comparison of experimental observations and model predictions of in vitro nucleosome occupancy on Saccharomyces cerevisiae chromosome 11. The experimental observation curve (green) and HDGS-Net prediction curve (orange) show high consistency within the 0-600,000 bp region. The x-axis represents chromosomal position, and the y-axis represents nucleosome occupancy.**


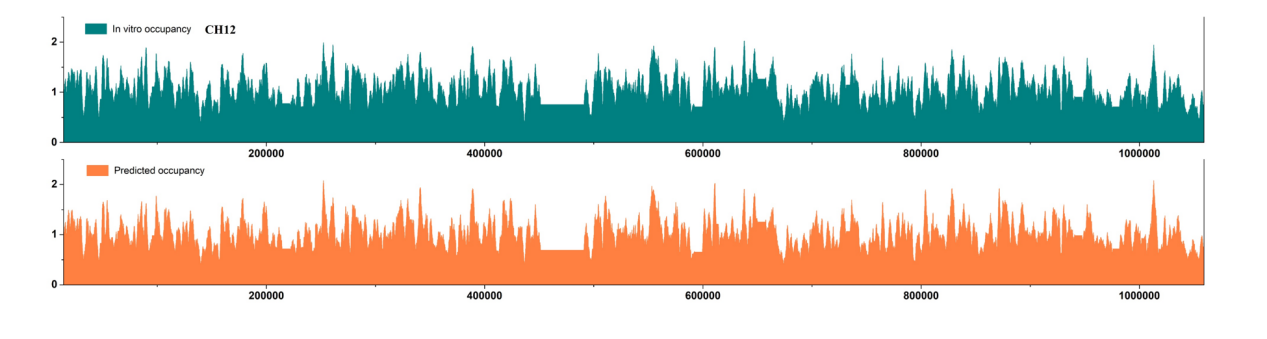


**Figure S11. Comparison of experimental observations and model predictions of in vitro nucleosome occupancy on Saccharomyces cerevisiae chromosome 12. The experimental observation curve (green) and HDGS-Net prediction curve (orange) show high consistency within the 0-1,000,000 bp region. The x-axis represents chromosomal position, and the y-axis represents nucleosome occupancy.**


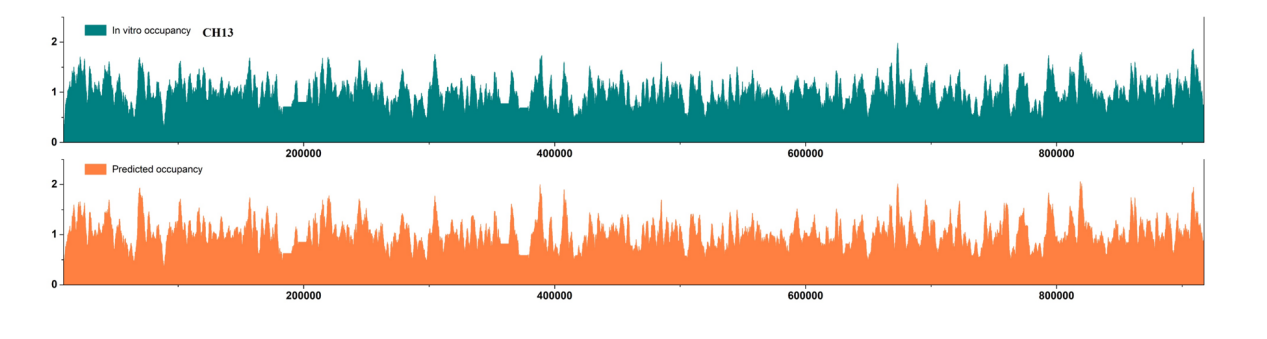


**Figure S12. Comparison of experimental observations and model predictions of in vitro nucleosome occupancy on Saccharomyces cerevisiae chromosome 13. The experimental observation curve (green) and HDGS-Net prediction curve (orange) show high consistency within the 0-800,000 bp region. The x-axis represents chromosomal position, and the y-axis represents nucleosome occupancy.**

**
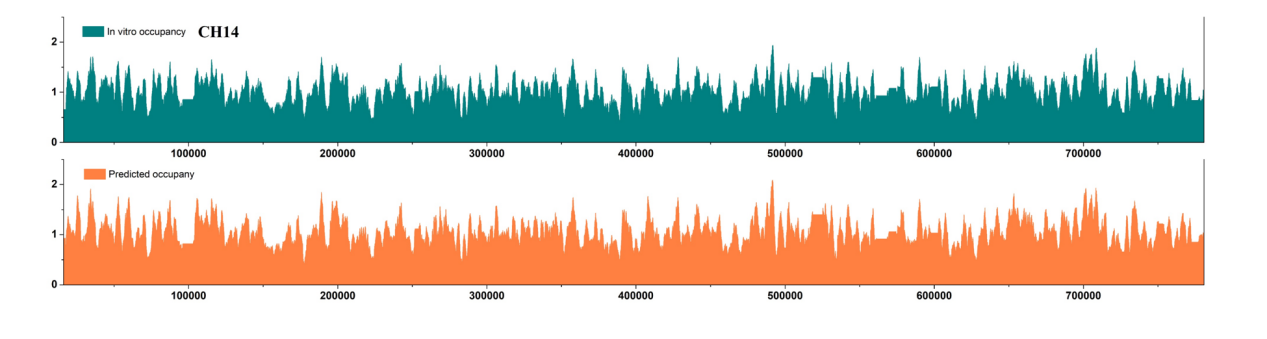
**

**Figure S13. Comparison of experimental observations and model predictions of in vitro nucleosome occupancy on Saccharomyces cerevisiae chromosome 14. The experimental observation curve (green) and HDGS-Net prediction curve (orange) show high consistency within the 0-700,000 bp region. The x-axis represents chromosomal position, and the y-axis represents nucleosome occupancy.**


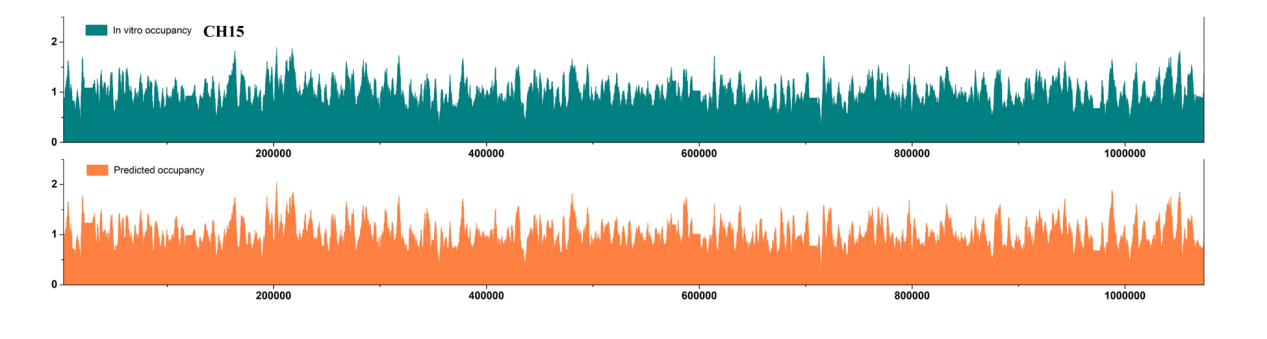


**Figure S14. Comparison of experimental observations and model predictions of in vitro nucleosome occupancy on Saccharomyces cerevisiae chromosome 15. The experimental observation curve (green) and HDGS-Net prediction curve (orange) show high consistency within the 0-1,000,000 bp region. The x-axis represents chromosomal position, and the y-axis represents nucleosome occupancy.**


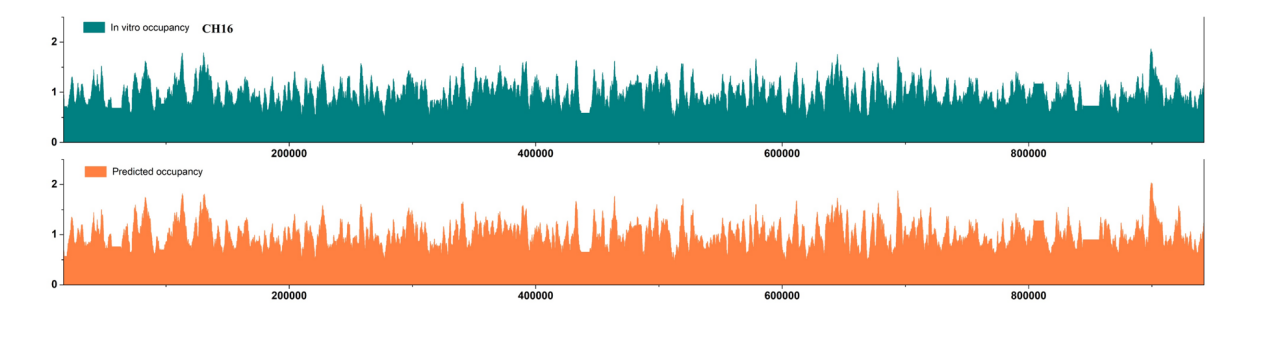


**Figure S15. Comparison of experimental observations and model predictions of in vitro nucleosome occupancy on Saccharomyces cerevisiae chromosome 16. The experimental observation curve (green) and HDGS-Net prediction curve (orange) show high consistency within the 0-800,000 bp region. The x-axis represents chromosomal position, and the y-axis represents nucleosome occupancy.**


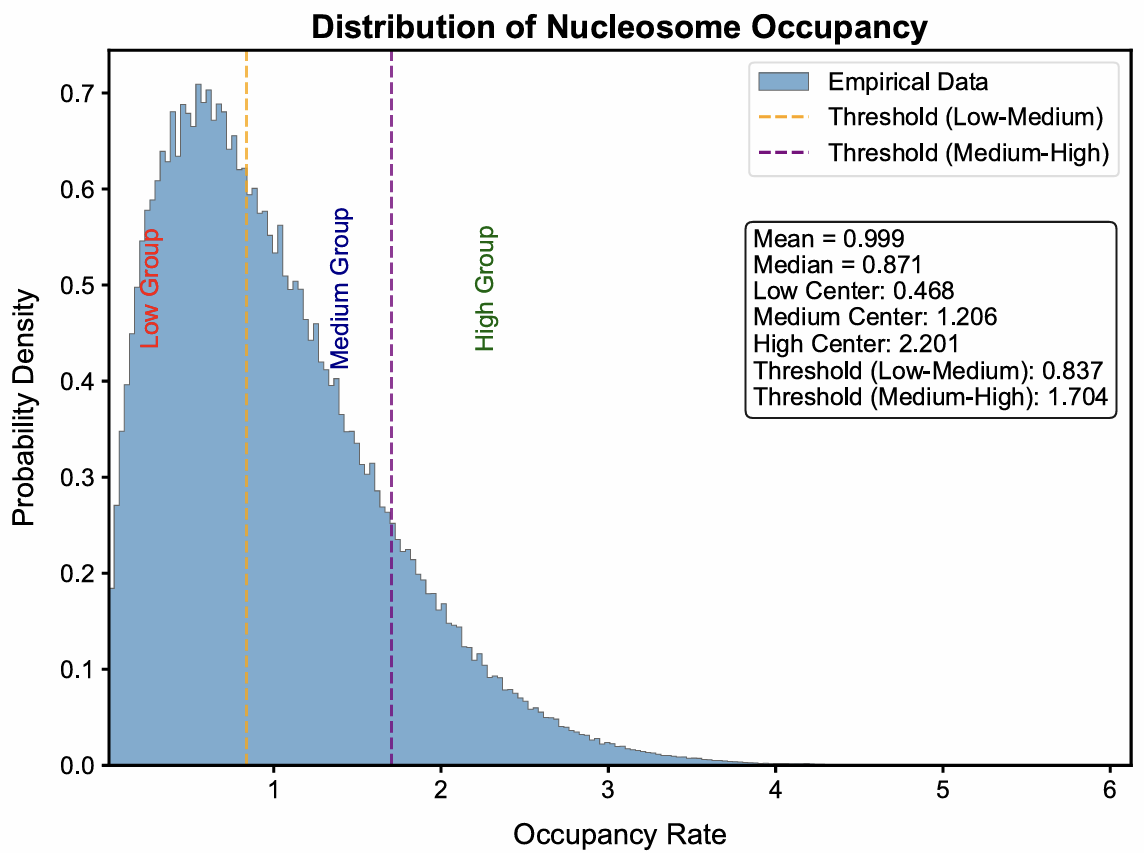


**Figure S16. Histogram of K-means clustering distribution for in vitro nucleosome occupancy, showing three distinct groups through threshold partitioning. The low occupancy group (49.05%) primarily distributes in the 0.2-0.5 range, the medium occupancy group (37.12%) broadly covers the 0.5-1.7 transition zone, and the high occupancy group (13.83%) concentrates in the >1.7 region.**


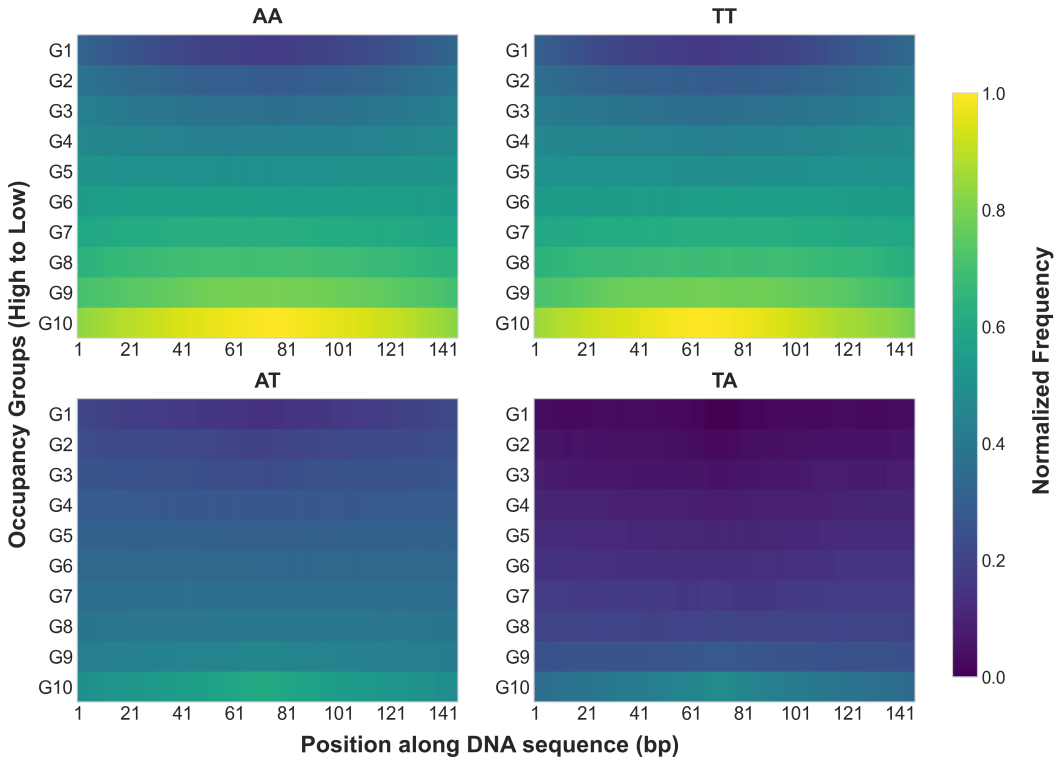


**Figure S17. Heatmap of AT-type dinucleotide distribution in genome-wide nucleosomes. AT-type dinucleotides (AA, TT) are enriched in the lowest occupancy group (G10).**


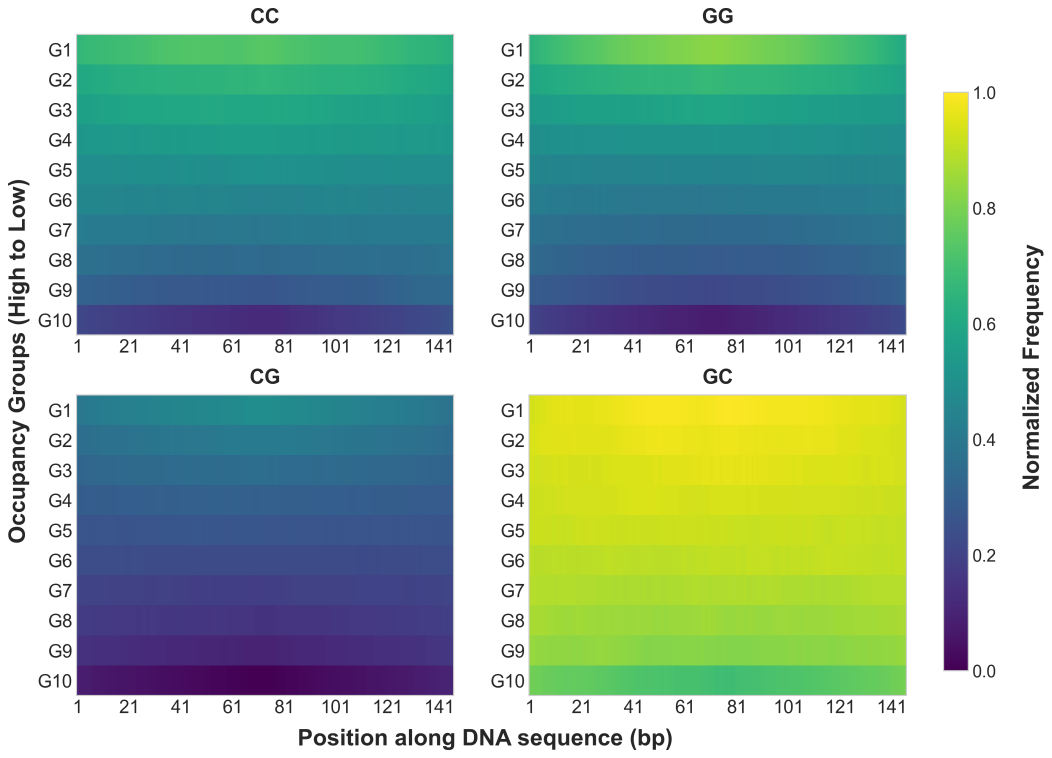


**Figure S18. Heatmap of GC-type dinucleotide distribution in genome-wide nucleosomes. GC-type dinucleotides are enriched in the highest occupancy group (G1), with GC dinucleotide frequency consistently exceeding other dinucleotides within the same category.**


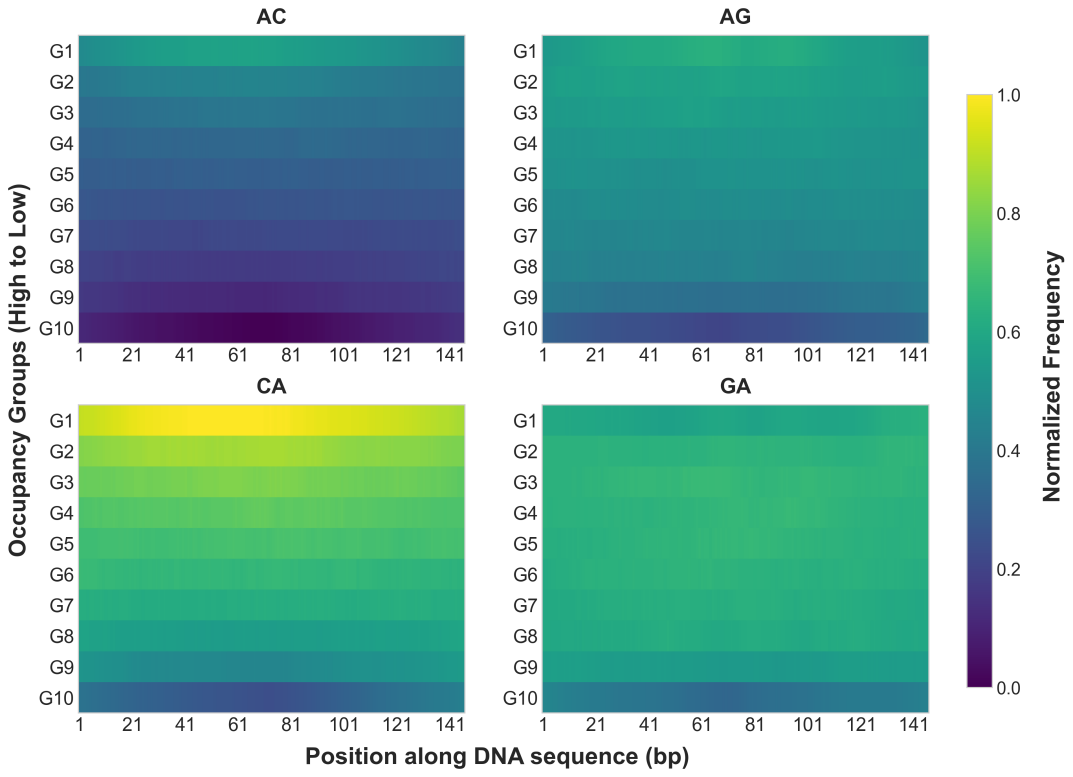


**Figure S19. Heatmap of mixed-type dinucleotide distribution in genome-wide nucleosomes. CA dinucleotides are enriched in the highest occupancy group (G1), and their frequency consistently exceeds that of other dinucleotides within the same category.**


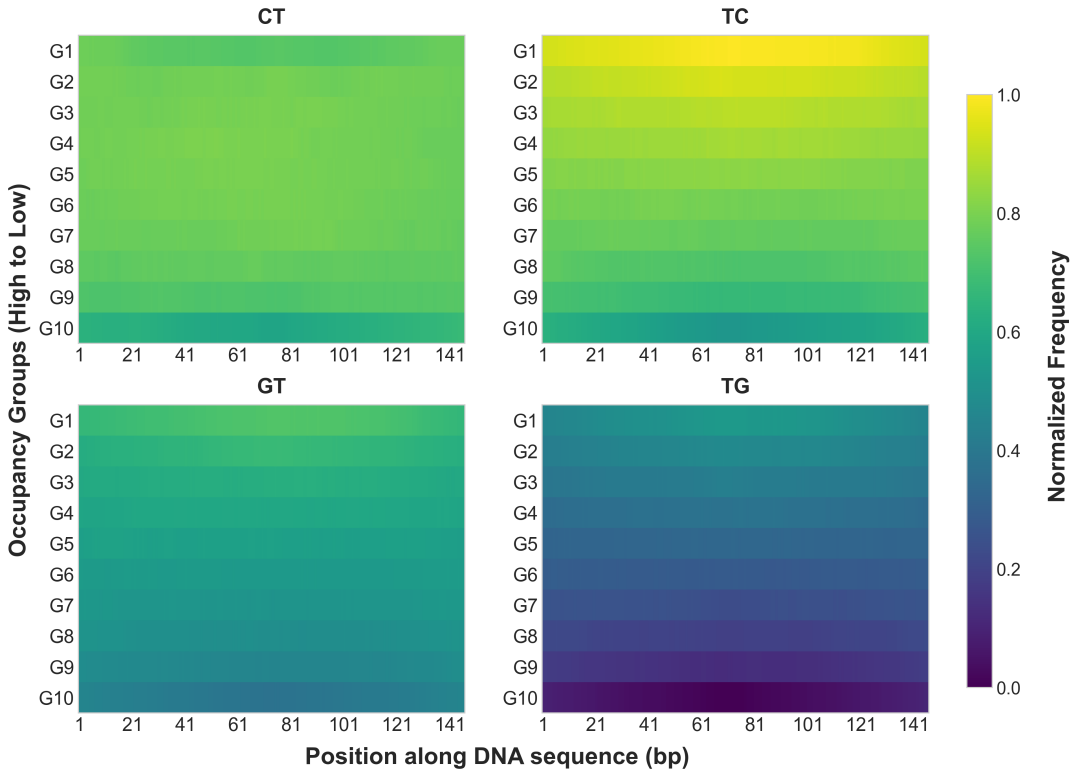


**Figure S20. Heatmap of transition-type dinucleotide distribution in genome-wide nucleosomes. TC dinucleotides are enriched in high occupancy groups, while TG dinucleotide frequency consistently remains lower than other dinucleotides within the same category.**


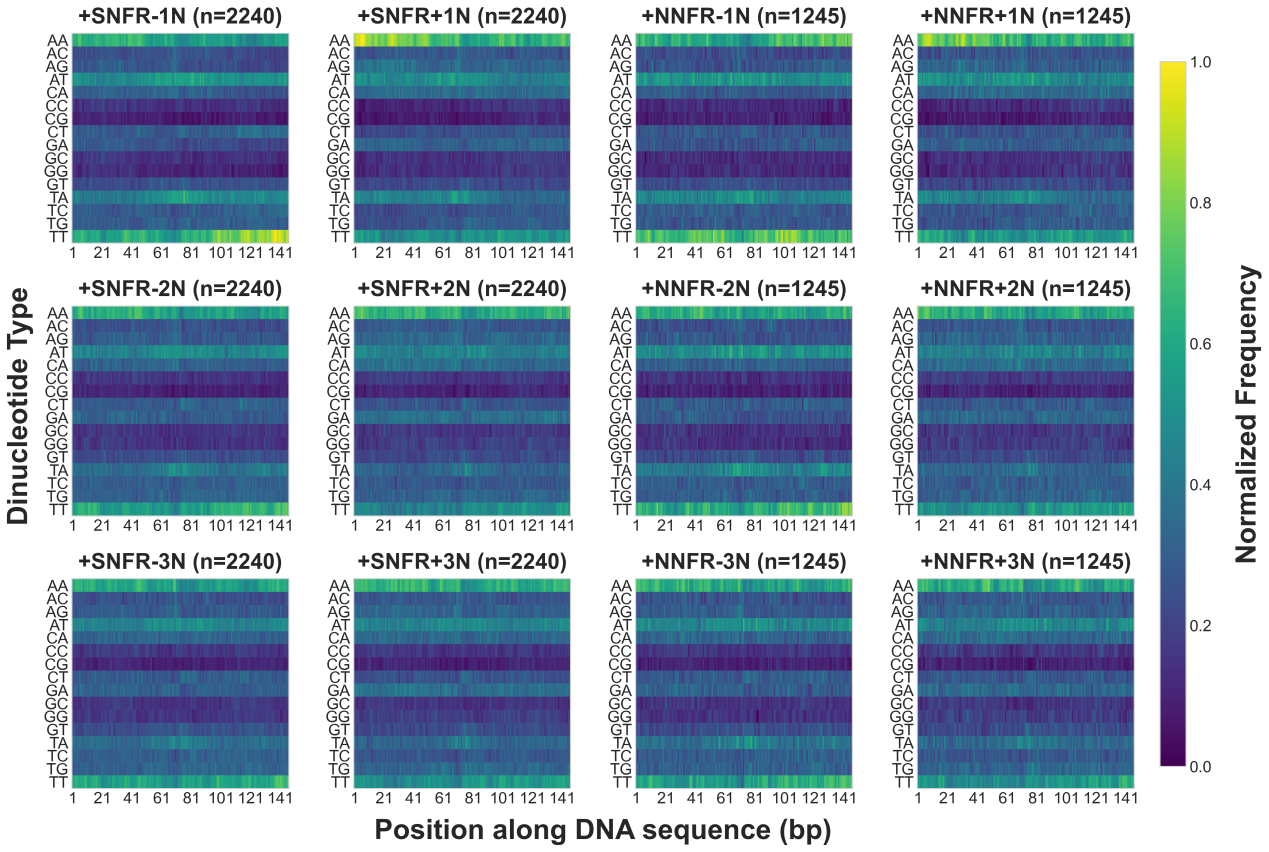


**Figure S21. Heatmap of nucleosome sequence features on the forward strand in TSS regions. SNFR and NNFR exhibit highly similar sequence features in cognate nucleosomes.**


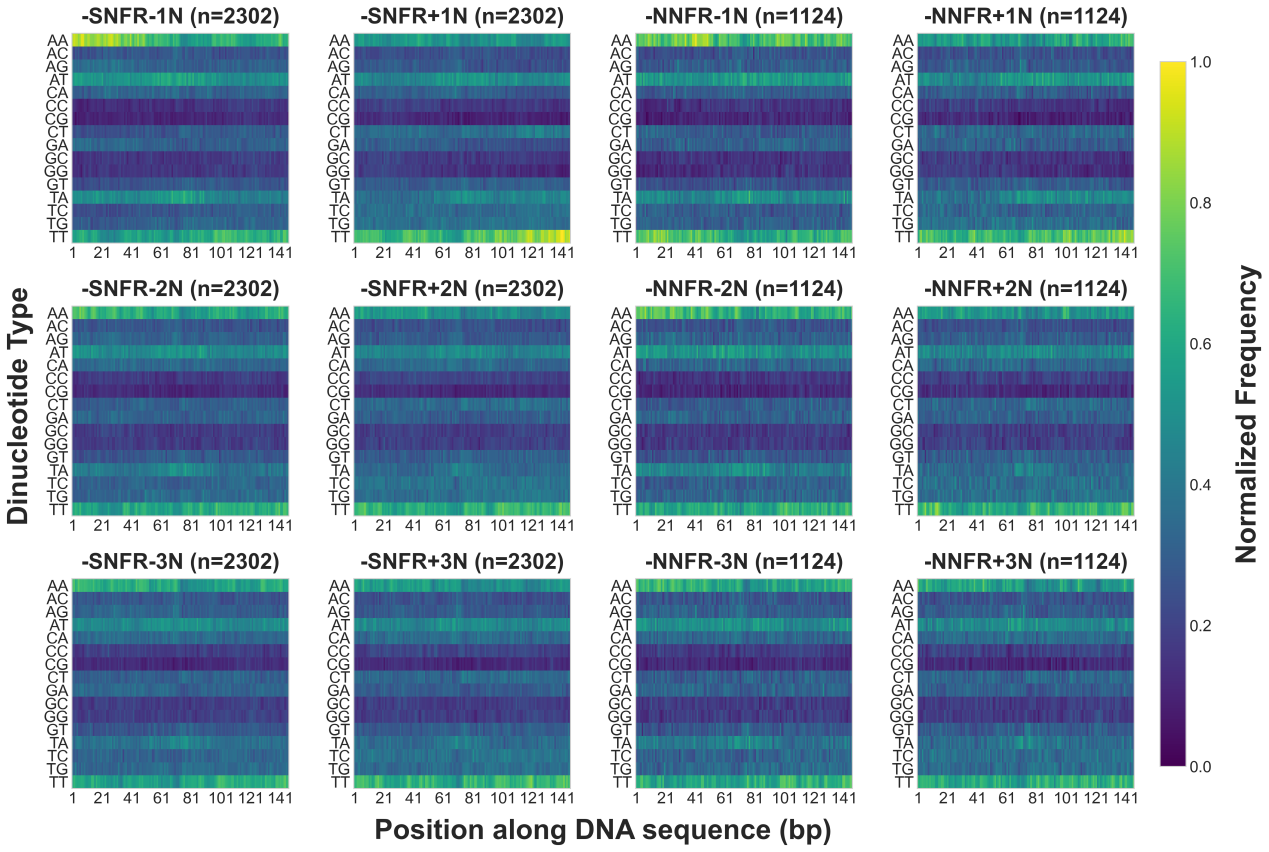


**Figure S22. Heatmap of nucleosome sequence features on the reverse strand in TSS regions. SNFR and NNFR exhibit highly similar sequence features in cognate nucleosomes.**


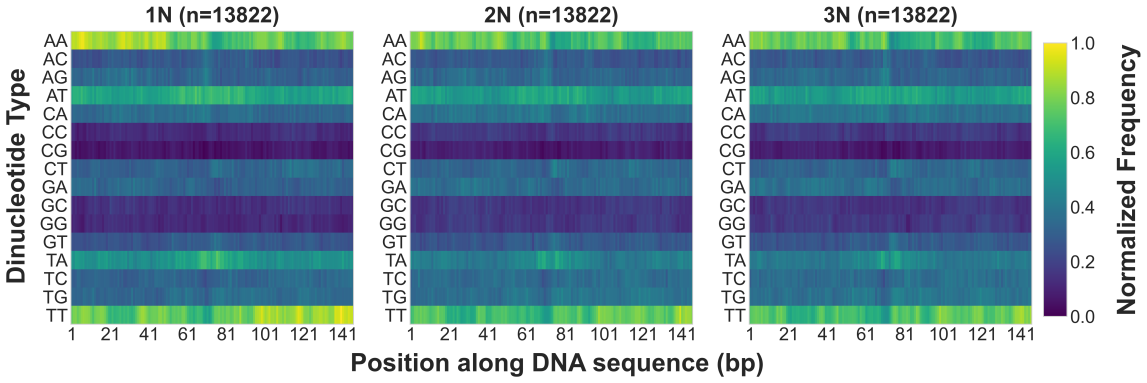


**Figure S23. Heatmap of nucleosome sequence features across symmetric groups in TSS regions. Sequence features show high similarity between groups.**
